# Adaptive Augmented Reality User Interfaces for Real-Time Defect Visualization and On-the-Fly Reconfiguration for Zero-Defect Manufacturing

**DOI:** 10.3390/s25092789

**Published:** 2025-04-28

**Authors:** George Margetis, Katerina Valakou, Stavroula Ntoa, Despoina Gavgiotaki, Constantine Stephanidis

**Affiliations:** 1 Institute of Computer Science, Foundation for Research and Technology—Hellas (FORTH), GR-70013 Heraklion, Crete, Greece; valakou@ics.forth.gr (K.V.); stant@ics.forth.gr (S.N.); gavgiotaki@ics.forth.gr (D.G.); cs@ics.forth.gr (C.S.); 2 Department of Computer Science, University of Crete, GR-70013 Heraklion, Crete, Greece

**Keywords:** augmented reality, smart manufacturing, defect detection, adaptive user interfaces, gesture-based interaction, context-awareness, Industry 4.0

## Abstract

Zero-defect manufacturing is one of the most promising strategies to mitigate failures within manufacturing processes, allowing industries to increase product quality efficiently and effectively. One of the challenges faced in the practical adoption of zero-defect manufacturing is that the most important aspect of manufacturing, people, is often neglected. Aiming to support shop floor operators, this work introduces a human-centric approach assisting them to become aware of defects in the production line and imminently reconfigure it. Our system comprises an Augmented Reality application that encompasses interfaces that dynamically adapt to different contexts of use and enable operators to interact naturally and effectively and reconfigure the manufacturing process. The system leverages the efficiency of the shop floor operators in monitoring and controling the production line they are working on, according to the task they are performing, and their level of expertise, to produce appropriate visual components. To demonstrate the versatility and generality of the proposed system we evaluated it in three different production lines, conducting cognitive walkthroughs with experts and user-based evaluations with thirty shop floor operators. The results demonstrate that the system is intuitive and user-friendly, facilitating operator engagement and situational awareness, enhancing operator attentiveness, and achieving improved operational outcomes.

## 1. Introduction

Industry 4.0 is revolutionizing the way we live and work through digital transformation [1]. Smart manufacturing is a new paradigm shift that intertwines advanced information and communication technologies with manufacturing, to enable accurate and effective real-time decision-making towards leveraging the production line [2]. Within this new paradigm, the goal of producing high-quality products with no defects has become crucial and led to the emergence of the concept of zero-defect manufacturing, which involves the use of advanced technologies and real-time data analytics to continuously monitor production processes and product quality, detect any abnormalities or deviations from the expected standards, and take corrective actions in real time to avoid defects [3]. Cyber–physical production systems, that is industrial automation systems that combine physical components, such as machines, robots, and sensors with digital components, such as software, play a crucial role in connecting equipment for intelligent decision support [4]. Smart manufacturing today comprises a diversity of technologies, such as Artificial Intelligence and Machine Learning, eXtended Reality, Internet of Things (IoT), and cloud computing, which act as the key enablers for realizing the five characteristics that classify a manufacturing system as smart: context-awareness, modularity, heterogeneity, interoperability, and compositionality.

Context-awareness is the most prominent of all the smart manufacturing characteristics for how the manufacturing ecosystem can operate in confluence with its environment, as it refers to the ability of a system, device, or application to perceive, understand, and utilize relevant contextual information, such as user location, time, activity, and environment [5,6]. The ability of a system to analyze and comprehend the user’s context of use enables it to adapt its behavior and content to better suit the user’s needs and preferences, leading to a more efficient, effective, and satisfactory User Experience (UX). In manufacturing, context-awareness facilitates real-time monitoring for performance optimization and risk detection, also improving safety [7,8]. It is thus evident that in recent years the research on smart systems in the manufacturing sector has been gaining momentum, aiming at enhancing manufacturing performance; nevertheless, currently, only a limited number of works investigate how context-aware systems can be applied in smart manufacturing towards zero-defect production lines. Current efforts also lack a comprehensive understanding of scalability and adaptability, partially meeting the needs of the overall manufacturing ecosystem. Additionally, important aspects such as interoperability and standardization that are necessary for seamless operation across diverse manufacturing environments and platforms are sparsely addressed by current approaches.

Situation Awareness (SA) is a critical concept in the context of factory operations, particularly in the era of Industry 4.0, marked by the increasing use of digital technologies [9,10]. SA refers to an operator’s ability to understand and anticipate events in their immediate environment and make effective decisions based on that understanding. Improving SA can enhance safety, productivity, and efficiency in factory settings. The ability to achieve high SA in challenging operational conditions poses a challenge for interactive systems supporting operators [11]. While SA refers to the human operator’s real-time perception and comprehension of their surroundings, context-awareness relates to the system’s ability to sense, interpret, and react to relevant contextual information. SA is also closely linked to the level of automation, which is intended to provide varying levels of assistance to operators depending on their tasks [12,13]. The use of different types of support is important in order to prevent the ‘out-of-the-loop’ performance problem, which occurs when operators are slow to intervene in situations where automation is not functioning properly [13].

Augmented Reality (AR) stands out in various fields for its ability to merge virtual elements with the real world, offering immersive experiences. Its benefits in interaction learning and efficiency have led to widespread adoption in education, healthcare, retail, tourism, entertainment, and Industry 4.0. Specifically, in the latter domain, AR enhances manufacturing processes by integrating advanced technologies [14,15,16]. In smart factories, AR aids human–machine interaction by providing real-time guidance to Production Line Operators (PLOs), improving their SA and decision-making while reducing errors [17,18].

In terms of interaction, gesture-based interaction in AR refers to the ability of users to interact with digital content in AR environments through natural and intuitive physical movements, enhancing immersion and safety in manufacturing [19]. Despite its popularity, building efficient gesture-based AR interactions requires a thorough knowledge of the user’s context. Current research on gesture-based interaction primarily focuses on elicitation studies to identify appropriate gestures for specific activities and the manipulation of user interfaces (UIs) to navigate through systems, rather than how users can reconfigure these systems. Adaptive AR systems utilizing gesture-based interaction can boost industrial efficiency and productivity. However, there is a limited exploration of these approaches for achieving zero-defect contexts, with a gap in designing context-aware systems adaptable to individual users’ contextual factors [20]. Although recent advancements in context-aware systems enable awareness of the operation context, operators still do not receive much beneficial feedback [20]. Addressing this gap could lead to the development of more efficient and personalized context-aware systems. In the manufacturing domain, developed applications typically involve training and guidance for workers, with the majority utilizing handheld devices that occupy the operator’s hands. Consequently, there remains a significant research gap in exploring the potential of gesture-based interaction and context-awareness in the manufacturing sector, specifically in the pursuit of zero-defect contexts.

In this research, we extend the scope of [11] by adopting the Human-Centered Design methodology specified by the ISO standard (International Organization for Standardization, 2019) and proposing a novel context-aware adaptive, gesture-based interactive AR system designed to assist PLOs during their daily tasks and enhance their SA. The proposed system is an extended library of adaptive user interface widgets that can be used as a development toolkit for AR applications designed for industrial environments. The system has been specifically engineered to aid operators in the surveillance of production lines by offering real-time alerts and information, as well as suggesting solutions and reconfigurations to direct the operators in addressing defects and errors. In this paper, “on-the-fly reconfiguration’ refers to the system’s ability to enable operators to dynamically reconfigure aspects of the production line—such as halting and resuming production or calibrating parameters in the manufacturing processes—through mid-air gesture input, without the need for direct physical interaction with machinery or control panels, as described in [21,22]. Based on the contextual information of the user, the system aids them in simpler and more complex tasks (e.g., monitoring, defect detection, and defect resolution) while they are still focused on their task at hand. The operator has an active role in the interaction with the system through the use of hand gestures, which enable not only navigation of the UI but also real-life actions.

The system leverages contextual information such as user expertise and production line characteristics to determine the type, location, and timing of information displayed in the user’s field of view, through Head-Up Displays (HUDs). This decision-making process is driven by a formal ontological model representing the manufacturing environment, encompassing entities like operators, tasks, gestures, and adaptive UI components, along with their relationships. A utility score is assigned to each adaptive UI component using Semantic Web Rule Language (SWRL) rules, assessing its contribution to the user’s SA. Additionally, SWRL rules infer on-the-fly system-reconfiguration activities that must be followed via the operator gestures considering current tasks, ensuring appropriate on-the-fly reconfiguration of the production line. The system was evaluated through user-based assessments in microelectronics, antenna and elevator-production lines, showing positive feedback on usability and effectiveness in enhancing SA.

This paper is structured as follows. Following this introduction, Section 2 reviews foundational concepts such as adaptive user interfaces, context-aware systems, Ontology-based modeling, and gesture-based interaction, highlighting existing gaps in the literature. Section 3 details the application of the Human-Centered Design process, including the elicitation of user requirements and the co-creation workshops conducted with stakeholders. Section 4.1 offers a comprehensive technical description of the adaptive AR system, including its decision-making pipeline, Rendering Framework, and UI component design. Subsequently, Section 5 presents the results from user-based studies conducted across three pilot manufacturing sites, focusing on usability, satisfaction, and situational awareness metrics. The paper concludes with a discussion of key findings, limitations, and directions for future work, ensuring a cohesive narrative that moves from problem identification to solution validation.

In this work, we developed the proposed approach in the context of the European research project OPTIMAI (https://optimai.eu/). The principal aim of this project is to enhance production efficiency, minimize defects, and improve training methods, with the main objective being to achieve zero-defect manufacturing within the context of Industry 4.0.

## 2. Related Work

This section discusses related work regarding the fundamental constituent components and concepts that the presented work explores and advances, including UI adaptation, approaches for enhancing SA, as well as Ontology modeling and reasoning. In addition, the methodologies for integrating gesture-based interaction in AR applications and proposed guidelines on the design of such applications are also considered within this section.

### 2.1. Adaptive User Interfaces

Adaptive UIs offer dynamic, personalized experiences by adjusting to users’ changing skill levels and the context of use, based on implicit cues gathered during interaction [23,24,25,26]. Ref. [27] define adaptive UIs as capable of dynamically changing based on user characteristics. UI adaptation has been defined as the quality that “… characterises software products that automatically modify (adapt) their interactive behavior according to the individual attributes of users (e.g., mental/motor/sensory characteristics, preferences), and to the particular context of use (e.g., hardware and software platform, environment of use)” [28] and was further classified into static (adaptability) and dynamic adaptation (adaptivity). The adaptability of a system is usually defined at the design phase, typically through exhaustively identifying all the potential different interactions that may lead to UI adaptation by collecting requirements from representative users [29]. However, this process almost always is time-consuming [30]. While many users appreciate not having to customize UIs themselves, their unique needs may not always be accounted for [31]. Adaptive UIs facilitate catering to diverse user needs, fostering universal accessibility through a Design for All approach [32]. Such adaptivity ranges from product- and environment-specific adjustments to generic solutions for universal user interfaces [33]. The literature reports various systems supporting adaptivity across domains like web applications, games, and intelligent environments, as well as platform-specific toolkits and design tools [34,35,36]. Comparatively, adaptive UIs outperform static and complex UIs by improving usability and user performance, as observed by [24]. Ref. [37] found also that they enhance task success, reduce unnecessary actions, and influence user choices. Ref. [38] show that even low-cost adaptive UIs can significantly boost user performance and satisfaction. However, ref. [39] suggests further refinement in the interaction between adaptive UIs and users is necessary for enhanced satisfaction.

### 2.2. Context-Aware Adaptive User Interfaces

Ref. [40] propose a context-aware online adaptation approach for designing Mixed Reality (MR) interfaces that dynamically adjust to the user’s context, incorporating user feedback and machine learning. The system comprises a user feedback mechanism capturing preferences and behavior, coupled with a machine learning system training a context-aware adaptation model. Demonstrated via a case study of an MR interface for furniture assembly, this approach significantly enhances user performance and satisfaction compared to non-adaptive interfaces. Ref. [41] investigate the utilization of AR as an interface to adaptive hypermedia systems, employing a prototype AR interface to the Interbook system for learning historical sites. Their study reveals that the adaptive AR interface is more engaging, immersive, and effective for learning compared to traditional web-based interfaces. Similarly, ref. [42] present an approach involving context markup and style mapping systems for AR content, showcased through a prototype AR system for urban tourism. By adjusting content and style based on user location and interests, the system enhances user experience and engagement. Ref. [43] describes a mobile AR architecture supporting learning across diverse contexts, incorporating sensors, an AR engine, and a context-aware system. Illustrated through a case study on marine biology learning, the architecture delivers personalized content in real time, enriching the learning experience and user engagement.

### 2.3. Model-Based Adaptive User Interfaces

Model-based adaptive user interfaces utilize models of user needs, preferences, and behavior patterns to dynamically adjust UI elements such as layout, content, and interaction style in real time [44,45]. Ref. [46] propose a framework-encompassing context, user experience, and adaptation models to personalize UIs based on current context and past interactions, as demonstrated in a medical self-assessment app case study. Ref. [47] introduce ActivFORMS, a model-driven approach facilitating self-adaptive systems with efficient runtime adaptation validated through an IoT security application. Ref. [48] present PersonisAD, an architecture for context-aware ubiquitous applications, emphasizing scrutability for user comprehension and model-driven development simplification.

### 2.4. Ontology-Based Modeling

An Ontology includes the series of heterogeneous things and classes that belong to a specific domain and the relationships between them in [49], whereas [50] describes an Ontology as “an explicit specification of a conceptualisation”. As mentioned in [51], Ontology refers to shared knowledge about concepts.

Ontology-based modeling involves representing context via the use of a variety of semantic Ontology languages and frameworks to model the given context. The World Wide Web Consortium (W3C) Web Ontology Language (OWL), the Resource Description Framework (RDF), and the Resource Description Framework Schema (RDFS) are all examples of semantic Ontology languages and frameworks that are used in Ontology-based modeling [52]. OWL (https://www.w3.org/OWL/) is the most popular semantic Ontology language because it offers a rich vocabulary that enables the representation of a wide range of concepts and relationships between them. The rich vocabulary and expressive power of OWL make it easier for machines to interpret and reason about the information on the web. The aforementioned languages and frameworks belong to the W3C’s Semantic Web Technology stack, which realizes the Semantic Web, an extension of the World Wide Web that enables better interpretation of the data that exist on the web [53]. Classes, attributes, and individuals make up the backbone of every Ontology [54]. A class refers to concepts that belong to a specific domain, an individual is an instance of a class, and a property defines the connection between two individuals or a person and a value. Some noteworthy Ontology-based context models that have been proposed in the literature are described briefly below.

Ref. [55] introduce a context-aware computing Ontology for modeling contexts and offering reasoning skills. Generic context ontologies include broad categories of information, whereas domain-specific context ontologies are tailored to particular areas of study. Basic context models, such as User, Space, Environment, Device, and Service are defined to record broad context details. The paradigm provides semantic logic that may be integrated with rule-based systems, allowing for inference methods to be used for verifying context inconsistencies and deducing implicit information. Since it is impossible for a single set of rules to cover every possible scenario, this study also introduces a rule-matching method that is based on the concept of semantic similarity.

Ref. [56] propose context-awareness meta Ontology modeling (CAMeOnto), which is a model employed by a reflective middleware for context-aware applications. This middleware is known as CARMiCLOC. It is written in the OWL language and organized into two tiers of ontologies: a top-level Ontology that applies to all domains; and a bottom-level Ontology that is particular to certain domains, as in [57]. Using the five Ws (i.e., who, when, what, where, and why), the first level of context specifies six types, namely user, activity, time, device, services, and location.

Towards Industry 4.0, ref. [58] proposed an Ontology-based context model for the industrial domain. The suggested method is based on the definition of context, which states that location, identity, time, and activity are the major context categories used to characterize a given entity’s condition. The proposed Ontology consists of smaller ontologies, general and more specific ones. In this sense, the Time Ontology, the Location Ontology, and the Sensor Ontology fall under the generic Ontology classification. On the other hand, domain ontologies define common ideas and characteristics for certain domains, which may be utilized in more specialized circumstances. In this category belong the Process, Resource, and Situation ontologies, since these ideas are important for describing industrial operations. Ref. [59] present an Ontology built according to a foundational Ontology (DOLCE), which has been deliberately impacted by concepts of natural language and cognition. The suggested Ontology is conceptually transparent and semantically explicit, two essential requirements for information exchange, sharing, retrieval, and re-usability. MANSON is another manufacturing Ontology described by [60], focusing more on the OWL representation of the model. Using an Intelligent meeting room as a use case, the Context Broker Architecture (CoBrA) project proposes a broker-centric agent-based architecture [61,62]. This architecture can be used for designing context-aware systems in smart spaces. The OWL language is used to create a set of ontologies for modeling, exchanging, and reasoning about context information. These ontologies describe suitable semantic concepts and relationships for describing physical places, time, people, software agents, mobile devices, and meetings. The context broker, which is an integral component of the architecture, is responsible for the following: (a) Maintaining a centralized Ontology-based context model, and sharing it across various devices, services, and agents of the intelligent space; (b) Reasoning over the stored contextual knowledge; (c) Obtaining contextual information from heterogeneous sources; (d) Protecting user privacy by enforcing privacy policies and procedures

### 2.5. Contextual Reasoning

Contextual reasoning, according to [63], refers to the process that *“is deducing new and relevant information to the use of application(s) and user(s) from the various sources of context-data”* (p.1). In context-aware systems, reasoning is necessary once context information has been modeled to infer new knowledge from existing knowledge. Ref. [64] discusses the use of ontologies in context-aware systems and how reasoning can be used to improve the accuracy and effectiveness of these systems through the proposal of an approach that combines Ontology-based reasoning and context-awareness to provide more accurate and personalized recommendations to users. The work also presents a case study on the development of a context-aware mobile application for tourism, which uses Ontology-based reasoning to provide personalized recommendations to users based on their preferences and context to demonstrate the effectiveness of the proposed approach. There are various context reasoning techniques. We will focus on Ontology-based reasoning, which is the approach followed in the current study.

The Ontology-based reasoning technique takes advantage of description logic, and Ontology-modeled data are used to reason about the context [57]. Ref. [65] propose an Ontology-based reasoning framework for context-aware applications. The framework uses ontologies to represent and reason about user and environment context, and generate recommendations and actions. The paper describes the architecture of the proposed framework, which includes several components such as a context model, a context reasoning module, and an action-generation module. The authors also provide a case study to illustrate the effectiveness of the framework in a smart home environment.

Semantic web languages, like SWRL, are used to implement it. The SWRL expresses rule-based first-order logic inference rules, written in terms of predefined OWL context information [66]. Other semantic web languages that are used to do this include RDF, RDFS, and OWL.

### 2.6. Gesture-Based Interaction for AR Systems

Gesture-based interaction in AR systems involves the use of hand and body gestures to control and manipulate virtual objects in the real-world environment. It is a natural and intuitive way of interacting with AR content and allows users to perform various actions such as selecting, moving, rotating, scaling, and manipulating virtual objects in real time. To enable gesture-based interaction, AR systems typically use a combination of sensors such as cameras, depth sensors, and motion sensors to capture and interpret user gestures [67]. These sensors can detect and track the position, orientation, and movement of the user’s hands and body in real time. Machine learning algorithms and computer vision techniques are then used to analyze and recognize these gestures and map them to specific commands or actions in the AR system [68,69]. One of the main advantages of gesture-based interaction in AR systems is its ability to provide a more immersive and engaging user experience. By using natural and intuitive gestures to control virtual objects, users can feel more connected to the AR environment and experience a greater sense of presence and agency [70]. This can be particularly beneficial in applications such as gaming, where the use of hand and body gestures can provide a more interactive and dynamic game-play experience [71]. Gesture-based interaction in AR systems also has potential applications in fields such as education, healthcare, and industry [72]. For example, in industry, gesture-based interaction can be used to provide workers with hands-free access to information and control machines or devices, improving safety and efficiency in the workplace [73].

Ref. [74] presents a set of guidelines pertinent to the interaction via gestures within AR systems. It elucidates that mid-air gestures are most appropriate for interactions in systems where the user’s primary attention is directed elsewhere. Simplified mid-air gestures are favored by users due to their ease of memorization and their capacity to enhance the immersive quality of the experience. Nonetheless, users exhibit limited patience for delays and errors in gesture recognition. Consequently, the adoption of a Human-Centered Design approach is advocated for determining the final set of gestures to be incorporated into a system.

### 2.7. Research Gaps and Contributions of This Work

After conducting a comprehensive state-of-the-art review, it has become evident that the application of context-aware systems in smart manufacturing to achieve zero-defect production lines is an area that requires further exploration. Despite the growing interest in smart systems for manufacturing performance enhancement, the existing research is limited, and the gaps are significant. There is a lack of understanding concerning how to develop scalable and adaptable systems that are user-friendly and meet the needs of human operators. Additionally, the absence of interoperability and standardization solutions hinders seamless operation across diverse manufacturing environments and platforms.

Context-aware systems have the potential to provide valuable insights into the production line’s performance and help operators identify and address potential issues before they become critical. However, further research is needed toward systems that are efficient in adapting to the individual operator’s needs and contextual factors. A human-centered context-aware adaptive system that provides personalized feedback and support tailored to the operator’s specific needs and context can significantly improve their overall performance and SA and reduce the likelihood of errors and defects in the production line. Furthermore, achieving zero-defect manufacturing is a critical goal for many industries, as it can reduce waste, improve customer satisfaction, and increase profitability. Therefore, developing a context-aware adaptive system that is human-centered can have a significant impact on achieving this goal and improving the overall performance of the manufacturing industry.

In the manufacturing domain, handheld devices that occupy the operator’s hands are the norm, and gesture-based interaction is an area that holds promise for enhancing operator–machine interactions. However, current research primarily focuses on identifying appropriate gestures for specific activities and navigating through systems, rather than exploring how users can reconfigure a system with them. Addressing these gaps in research could lead to the development of more efficient and personalized context-aware systems for manufacturing that can adapt to individual users and provide beneficial feedback. Such systems would significantly improve manufacturing processes’ efficiency, reduce defects and errors, and increase customer satisfaction, thereby positively impacting profitability.

Building upon existing research, this work attempts to cover these gaps and presents a step forward to the field of Smart Manufacturing Systems in Industry 4.0, particularly within the zero-defect context. It proposes a system that includes an extended library of GUI components designed according to the Human-Centered Design principles, which can be used to develop targeted applications for manufacturing plants. The primary aim of the proposed system is to assist shop floor operators by providing real-time information regarding detected defective products or predicted defects [22,75,76]. Furthermore, the system offers actionable insights to prevent further problems and handle the issues efficiently. The system also supports the operators in activities inside human-operated production lines in order to minimize the potential defects. The use of a Human-Centered Design methodology has numerous benefits, two of which are increased user satisfaction and improved usability. By involving users in the design process and prioritizing their needs and preferences, Human-Centered Design can lead to higher levels of user satisfaction and engagement. Additionally, the evaluation of adaptive intelligent systems poses several challenges in terms of effectiveness and efficiency, especially considering their dynamic nature [77]. Toward this direction, this work applied a two-phased evaluation approach involving both experts and end-users, aiming to predict the learnability of the system but also assess it under different operational conditions and contexts.

The UI of the system has been designed to adapt to contextual factors, such as the user’s level of experience, the specific production line they are occupied in, and the task at hand. This adaptivity can provide the operator with customized and relevant information based on their current task and environment, allowing them to work more efficiently and effectively. Secondly, it can assist the operator in making informed decisions by presenting relevant data and insights in real time. Additionally, it can improve safety by providing warnings or alerts based on the current context, such as potential hazards or deviations from standard operating procedures. Such a system can help reduce errors and improve quality by guiding the operator through complex or critical tasks, ensuring that they are performed correctly and consistently. The system’s context-aware adaptivity is achieved through Ontology modeling and reasoning. Using ontologies to reason about context can lead to more accurate decisions and increased flexibility by adapting to changing contexts and requirements, enabling systems to quickly respond to new situations. Ontologies provide a common language and framework for representing data, which can improve interoperability between different systems and devices and knowledge sharing and collaboration among stakeholders, trying to ensure that everyone involved in the system has a common understanding of the context and how the system should respond. In this respect, the current work contributes an Ontology for modeling the industrial environment, that can be used by adaptive context-aware applications developed for this domain.

Furthermore, the proposed system supports real-time reconfiguration of production line operations through mid-air gesture input. Unlike conventional approaches that limit operator interaction to simple UI navigation, the system introduced in this work uses semantic reasoning to interpret gestures within their contextual setting. This allows operators to interact with the system hands-free, and also trigger high-level actions such as halting the production line, switching to inspection workflows, or initiating corrective procedures. By using a second set of SWRL rules, the system determines whether a gesture is valid within the current state and selects the appropriate reconfiguration action accordingly. This capability empowers operators to intervene dynamically and safely in the manufacturing process, enabling more adaptive, responsive, and efficient production environments.

Another contribution of this work is that the proposed library is versatile and can be used in applications of diverse kinds of production lines. Currently, a development toolkit has been used for the development of three applications for three different pilot sites. These applications have been designed to operate seamlessly with AR glasses, which offer a hands-free experience to the operator, allowing them to interact with the system without the need for handheld devices. The system allows the operator to perform other manual tasks concurrently while utilizing it, without requiring them to use their hands to operate the system, thus enabling hands-free operation. This goal is also achieved with the integration of mid-air hand gestures, which enable the operator to navigate and interact with the system in a natural and intuitive manner. A specific set of gestures has been designed for the system with the knowledge acquired from the literature review and each gesture corresponds to specific actions maintaining consistency throughout the system. The gestures apart from the manipulation of the UI can also evoke changes and reconfigurations to the production lines.

## 3. Methods

This work adopts the Human-Centered Design approach for the design and development of interactive systems, as has been specified by the ISO standard (International Organization for Standardization, 2019). This approach prioritizes the active involvement of representative end-users and stakeholders throughout the entire development life-cycle, resulting in interactive products that are usable and provide a high-quality user experience. The iterative process of Human-Centered Design involves four main activities: understanding and specifying the context of use, specifying the user requirements, producing design solutions and prototypes, and evaluating the designed solutions. By following this approach, the design takes into account the needs, preferences, and behaviors of the users as well as the context in which the system will be used. The Human-Centered Design approach aligns with best practices in human factors and ergonomics research, emphasizing the importance of designing for the user and their environment to optimize performance, safety, and satisfaction.

### 3.1. Understand and Specify the Context of Use

In the first phase of our approach, we established the context of use through the facilitation of three co-creation workshops with experts, stakeholders, and workers from the three specified case studies [78]. Co-creation is “a creative process that taps into the collective potential of groups to generate insights and innovation. Specifically, it is a process, in which teams of diverse stakeholders are actively engaged in a mutually empowering act of collective creativity with experiential and practical outcomes” [79]. Co-creation is a powerful innovation tool pivotal to the employed Human-Centered Design approach, which can lead to valid, rich, and diverse conclusions within a research context [80]. We were able to gain insights into various contextual factors regarding the system: the users, their characteristics, their needs, and their goals and tasks as well as the environment of the system. Key findings for the context of use are as follows: (i) the system is designed for experienced or moderately experienced production line operators who need hands-free interaction to avoid interference with their work; (ii) the users should be comfortable and safe on the shop floor; (iii) the system should assist in daily tasks such as monitoring production lines, detecting and addressing defects, and enhancing context-awareness; (v) it is intended for use in various manufacturing plant environments.

### 3.2. Specify the User Requirements

Furthermore, by defining the user needs we were able to specify the functional and non-functional requirements that should be addressed by the system. According to the Human-Centered Design, this process must be extended to explicitly state the user requirements in relation to the context of use and business objectives. If the studied system may affect organizational practice, the development process should involve organizational stakeholders as well, providing insights on both technical and organizational aspects. For that matter, in all the co-creation workshops, managerial staff participated to ensure the smooth operation of the production lines. The operators’ and other stakeholders’ needs were identified considering the context of use, including what is needed to achieve their goals and overcome any constraints imposed by the context. The workshop results were analyzed in a structured and organized manner, and the results of this analysis were used as the basis for identifying and defining the list of the system’s functional and non-functional requirements.

The user requirements were elicited through three co-creation workshops, carried out online, involving stakeholders from three different production lines (microelectronics, antennas, and elevators), involving fifteen participants of different responsibilities, such as site managers, shop floor operators, designers, and developers. To steer the discussion and conduct a systematic analysis of all potential requirements, two methods were followed:

1. Hierarchical task analysis [81], systematically analyzing all the tasks that an operator could carry out using the proposed system on the shop floor.
2. Low fidelity mockups [81], showcasing the pertinent mockup for each task step, to the participants, and discussing regarding the information that the system should deliver and the adopted UI approach.

The workshop results were analyzed and a list of 21 requirements was developed, organized into four distinct categories, as follows: general requirements that are common for all the production lines, and requirements concerning the microelectronics-production line, the antenna-production line, and the elevator-production line. The general requirements are as follows: (i) the system should be able to support the fast and accurate interaction of the operator and machine; (ii) the system should be able to display information in the users’ field of view through binocular smart glasses lenses; (iii) the system should provide intuitive visual analytics on the workers’ AR glasses concerning the quality level of production; (iv) AR smart glasses should be used to present the analytical results from monitoring and inspection and visualize the analysis results depending on the viewpoint of the operator; (v) decision-making should be supported by the aggregated analytics results and suggestions; (vi) the Decision-Support System should import data from the AI-based tools and the Human–AI collaboration mechanisms; (vii) the Decision-Support System should receive data that will be processed in real time and integrate assistance solutions for shop-floor operators in decisions related to the detection and anticipation of anomalies in the manufacturing processes.

### 3.3. Evaluate the Designed Solutions

User-based evaluation is a crucial aspect of a human-centric system implementation, requiring the assessment of design concepts based on the perspective of end-users. Such evaluation should be conducted as early as possible in the design process to obtain a comprehensive understanding of the users’ needs. Despite ergonomic design guidance providing useful support to designers, user-based evaluation remains an essential element of Human-Centered Design, given the complex real-world usage of products, systems, and services. While evaluation by users on early system prototypes, for user-based testing, is preferred, it may not always be practical or cost-effective at every stage of the project. In this case, an alternative evaluation method was selected in the early stages of the design process, that of cognitive walkthrough [82], to evaluate design solutions with experts and determine aspects of the interface that would be challenging to users. Therefore, although not involving directly end-user hands-on assessment, this method is centered on how users will experience the system. At a later stage, having eliminated all identified usability problems, in situ evaluations with representative end-users were conducted at the studied production lines. This approach for early user assessment of the system served several purposes, including collecting new information about user needs, providing feedback on design strengths and weaknesses from the user’s perspective, assessing whether user requirements have been met, and establishing baselines or making comparisons between designs. The processes and results of the evaluations are thoroughly described in Section 5.

## 4. The AR Library for Real-Time Information Visualization of Defect Detection in Production Lines

As mentioned, the present work builds upon previous research [11] and uses Ontology-based reasoning and combinatorial optimization to improve users’ SA so that they can in a timely manner be aware of a system malfunction. This approach involves dynamically adapting the UIs in real time while considering the current context of use. To this end, the system can present relevant information through AR headsets during runtime. For the effective visualization of information, the specific methodology involves decision-making on what content to display, when to show it, where to place it on the screen, and how to present it effectively. More specifically, these decisions are made by the Decision Maker module, which can be broken down into three components. The Ontology Model component represents the knowledge of the supported adaptive UI elements and the contextual factors (user, activity, environment, device), and dynamically stores data regarding the current context (e.g., user’s profile, operation task, AR glasses resolution, etc.). The second component is the SA Reasoner, which evaluates the suitability of each element in the adaptive UI to improve the user’s SA by assigning it an SA score. The assessment is based on the Ontology Model, current context, and domain knowledge, represented by Ontology rules. The UI Optimizer determines the best adaptation of the UI, including selecting elements, their presentation, and placement for display. The decision is influenced by the SA scores given by the SA Reasoner, as well as visualization and placement constraints, such as size, shape, and current context from the Ontology model. The Rendering Framework then displays the adapted GUI elements. This approach is designed to assist users in making quick and well-informed decisions by dynamically adapting AR display in real time while considering various factors such as user characteristics, environmental and system factors, and the current task at hand.

Laid on the foundation of the aforementioned approach, a new system for the manufacturing sector was developed, aiming at enhancing the shop floor operators’ SA. Since the aforementioned study was intended for use in public security, the requirements and the context of use are different from the industrial settings. Specifically, a Production Line Operator (PLO) needs to be highly aware of the environment, continually monitoring the equipment and production line workflow to ensure that everything is operating correctly and also to identify any potential issues before they can lead to defects. Additionally, a PLO should be familiar with quality-control procedures and standards, and be able to follow them precisely to prevent any defects from occurring. Apart from preventing defects through vigilance and proactivity, PLOs should promptly and efficiently address any emerging issues. This may involve stopping production, resolving a problem, and only recommencing production after complete issue resolution. The proposed system has been implemented to meet the specific requirements of a manufacturing environment, as discussed in the previous section, with the intended end-users being PLOs. To achieve this, a new ontological model was specified, and new GUI elements were designed and implemented. New reasoning rules for determining the utility scores of the visual components of the system have been specified considering factors such as the production line in use, the specific task being performed, and the operator’s level of expertise, aiming at enhancing the PLOs’ SA. Each production line and task supports different types of information that can be visualized to the user. In contrast to the previous approach regarding security, which only requires urgent information alerts to be displayed during emergencies at positions that do not distract the field of view of the users, considering that they act under high stress and should be very focused on suppressing criminal activities, PLOs in a manufacturing environment may need to have more information in their field of view when no emergency is present. To address this requirement, the decision-making module has been extended to calculate and select more possible positions for widgets on the AR display.

A pivotal feature of the discussed system is the incorporation of gesture-based interaction, based on a gesture vocabulary. The vocabulary comprises a set of intuitive, easy-to-learn gestures that enable users to navigate through the system and modify it. The integration of gesture-based interactions and their corresponding system responses into the Ontology model is achieved by leveraging Ontology reasoning techniques. This approach aims to restrict the execution of specific system actions to be contingent upon the performance of particular hand gestures by the user under predefined contextual circumstances. The following sub-sections provide an in-depth analysis of all the components of the system.

### 4.1. System Architecture

**Figure 1:**
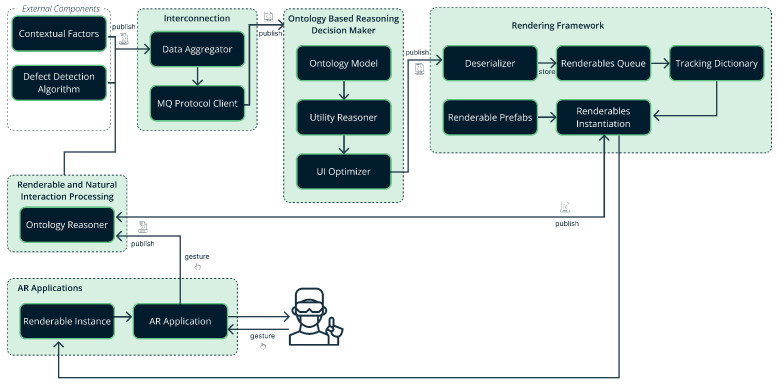
System’s architecture.

The system is built on a loosely coupled architecture of interconnected components operating in a specific pipeline. The system architecture comprises five distinct component groups, each subdivided into smaller components: Interconnection, Ontology-Based Reasoning Decision Maker, Rendering Framework, Renderable and Natural Interaction Processing, and AR Applications [83]. Figure 1 illustrates the system architecture showing the five main component groups and their data exchanges. Arrows indicate message flow or data triggers between modules and external components.

- Interconnection Component: The Interconnection Component consists of the Data Aggregator module and the MQ Protocol Client module. The Data Aggregator is responsible for forming a JSON message with all the collected information gathered from the real-time machine learning mechanisms monitoring the production line [84], for the Decision Maker. The message includes the contextual factors of the user and the HMD and the kind of information that needs to be displayed. The information to display varies depending on the reaction that the Data Aggregator receives from the Renderables and Natural Interaction Processing component which is explained in detail below. The Data Aggregator also communicates with external black box computer vision algorithms for detecting items and defects in specific cases and adds this information to the message when needed. The MQ Protocol Client module facilitates the exchange of JSON messages between the Data Aggregator and the Decision Maker.
- Ontology-Based Reasoning Decision Maker: The Decision Maker has to process the received information and select the optimal widgets to be displayed for maximal PLO situational awareness. The Decision Maker consists of the Ontology Model, the Utility Reasoner, and the UI Optimizer. The Ontology Model represents the knowledge surrounding the GUI widgets library, the entities involved in the use of an application integrating the library and their properties. These entities are namely the user, which is a shop floor operator, the production line in which the system would be incorporated, the operator’s tasks that the library can support, the gestures that the library supports, and the possible reactions. The Utility Reasoner based on the Ontology model dynamically quantifies each GUI element’s suitability for display (its utility score), in terms of improving the SA of the user. The scores obtained from the Utility Reasoner are passed to the UI Optimizer, which designs the GUI layout.
- Rendering Framework: The information generated by the UI Optimizer is passed to the Rendering Framework in the form of JSON messages with the MQ protocol. The information is received by the Deserializer component that deserializes the JSON message, transforms the deserialized information, and stores it in a format that can be easily processed. The information that is deserialized includes all the renderable elements that need to be visualized, their position on the application UI, their size, their Level of Details (LoD) and other properties. This information is stored in a Queue structure, the Renderable Queue. Furthermore, the Tracking Dictionary assigns unique IDs to the elements that are visualized to keep track of them throughout the next frames storing them in a Dictionary structure. When the elements are to be displayed, the Renderable Instantiation function is called up. This function instantiates adaptive UI widgets that represent each selected element for visualization.
- Renderable and Natural Interaction Processing: This component is responsible for handling the gestures performed by the user. At first, the component is notified by the MQ protocol that a gesture has been detected. The component includes an Ontology Reasoner for deciding, depending on the current visualized elements and other contextual factors, whether and which reaction should be triggered based on the received gesture. Then it informs the Data Aggregator regarding the reaction or else informs the Renderables Instantiation function to continue the visualization.
- AR Applications: The library is developed using Unity3D (https://unity.com/), compiled into an APK file, and can be installed on the AR glasses ready for use. Finally, this pipeline is repeated while the application runs on the AR glasses.

### 4.2. Interconnection Component

The Interconnection Component handles the connections between all the components for the visualization of the information and the external services. It consists of the Data Aggregator and the MQ Protocol Client.

#### 4.2.1. Data Aggregator

The Data Aggregator component is a standalone Python, tested with version 3.11.7, program that is responsible for managing and filtering the information provided to the Decision Maker. More specifically, the Data Aggregator contains information regarding the context of the application’s use. It also communicates with the Renderables and Natural Interaction Processing component to receive the reaction that is triggered with every gesture. Based on this reaction, it forms a JSON message including the contextual information to send to the Decision Maker. The Data Aggregator also receives messages in JSON format regarding the detected defects and items by external Computer Vision algorithms.

#### 4.2.2. Message Queuing Protocol Client

At present, communications inside the proposed pipeline are facilitated via a locally served message broker using the RabbitMQ (https://www.rabbitmq.com/) message-oriented middleware solution. This message broker employs microservices-based asynchronous communication, utilizing the Advanced Message Queuing Protocol (AMQP). RabbitMQ was chosen for its capability concerning simple AMQP client and server architecture with publish/subscribe communications and work queues. Basically, using RabbitMQ, clients transmit messages to a server (local broker, in this version). These messages are broadcast to an exchange or queue for other clients to consume. Queues store each message until the intended client consumes it. A distinct queue may be built per client, enabling clients to consume separate copies of messages at their own leisure, without compromising the functioning of other clients.

### 4.3. Ontology-Based Inference and Decision Maker

The JSON message sent by the Interconnection Component is then processed by the Decision Maker, which infers the most suitable GUI elements to visualize, calculates their relevance based on the current context, and determines how they should appear on the screen. This logic is explained in the following subsections.

#### 4.3.1. Ontological Model

**Figure A1:**
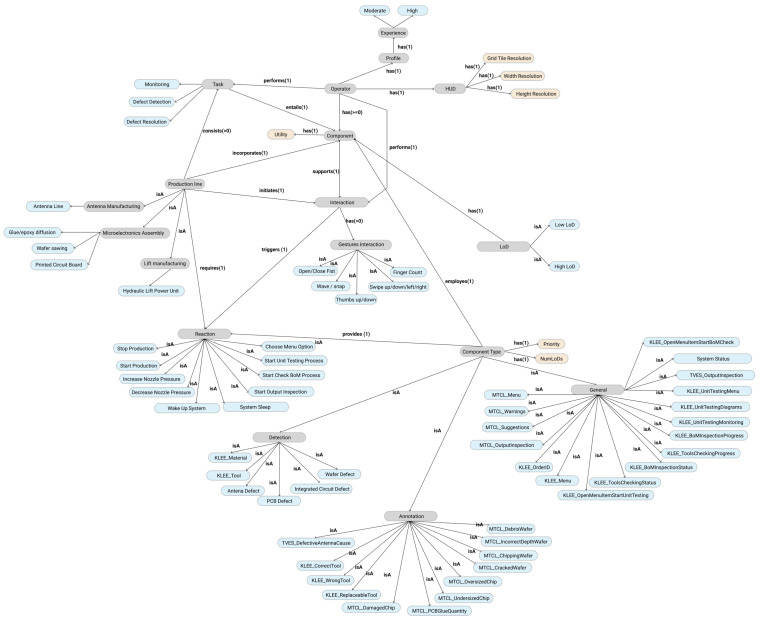
Detailed view of the ontological model.

In order to capture the intrinsic conceptual structure of the system’s operational environment, a knowledge representation of the connections and reliances among its various components needs to be specified. For this purpose, an ontological model is proposed. The designed Ontology model (Figure A1) is divided into two parts.

The upper part models the user—specifically, the PLO—and associated entities, such as their experience level (‘High’, ‘Moderate’), the AR HUD they are equipped with, its configurations, and the tasks they may perform (e.g., monitoring, defect detection, defect resolution). Each GUI component that can be displayed on the HUD is also represented in this part of the model, including attributes such as utility score, LoD—which can be low or high—and priority. These attributes are used to reason about component relevance and selection depending on the context of use.

The lower part of the Ontology focuses on interactions, supported GUI components, and the logic behind triggering system reactions. The operator’s gestures (e.g., *Open/Close Fist, Wave, Swipe, Thumbs-Up/Down*) are modeled as interaction types that, under specific conditions, trigger corresponding system reactions (e.g., *Stop Production, Start Output Inspection, Check Bill of Materials*). These reactions are influenced by the current task, line status, and detected context.

The Ontology also models the three types of supported production lines: (a) microelectronics assembly, (b) antenna manufacturing, and (c) lift manufacturing. Each production line includes a defined set of tasks. For instance, in the microelectronics assembly line, operators may work on the *Glue Diffusion, Wafer Sawing, or PCB Routing* lines. Tasks like *monitoring* involve the identification of defects, while *defect detection and resolution* tasks involve taking corrective actions—often requiring operator-triggered reactions such as recalibrating machine parameters or stopping production.

Furthermore, the Ontology model’s supported GUI Components are divided into three main disjoint component categories:

- The first category is the ‘Detection’ category, which corresponds to components used to highlight identified defects or objects of interest in the scene (e.g., Wafer Defect, Antenna Defect, KLEE_Tool).
- The second category is the ‘Annotation’ category, which includes components that display contextual details about the detected item, such as tool status (CorrectTool, ReplaceableTool, WrongTool).
- The third main component category is the ‘General’ category, which consists of components that are not linked to a specific detection but provide system-level or process-level information (e.g., Warnings, Suggestions, Menus, System Status).

Each Component Type may support one or more LoDs, allowing the system to adapt the presentation complexity based on the user’s context or task. The attribute hasNumberOfLoDs specifies the number of LoD variants each component supports; in the current implementation, this ranges between 1 and 2.

#### 4.3.2. Utility Reasoner

In accordance with the SA score in [11], a utility score needs to be assigned to each UI Component in order to define its suitability for display in the operator’s field of view. The utility score indicates how appropriate a component is to be presented in the UI and, more specifically, how much it enhances the SA of the user compared to the other components, considering the contextual information that is currently available. Based on this Ontology model, the Utility Reasoner is responsible for calculating this utility score depending on its Component Type and LoD.

**Figure 2:**
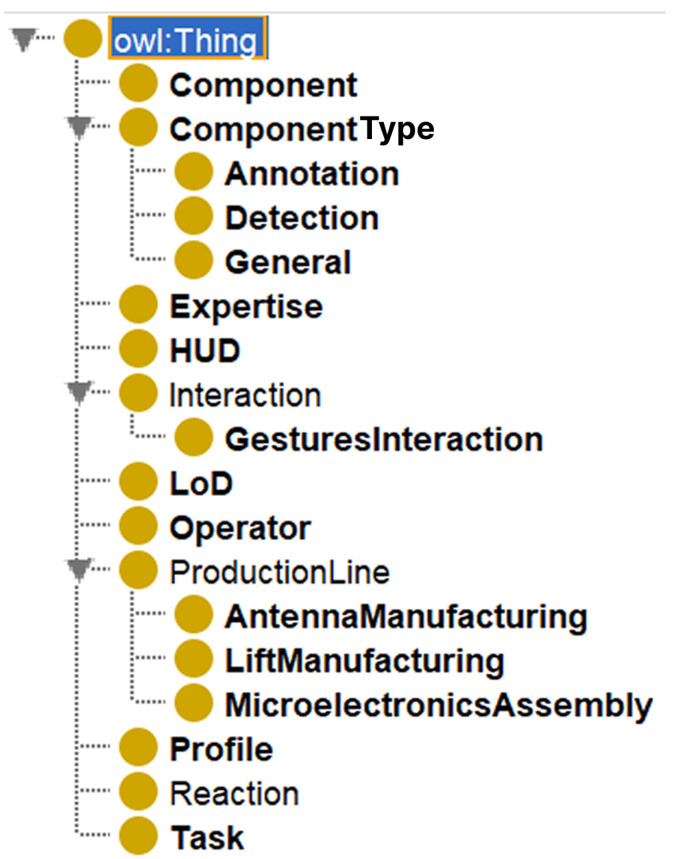
Class hierarchy of Ontology classes in Protégé.

The steps followed for the development of the Ontology are described next. Initially, the fundamental notions of our application domain were captured via the hierarchical definition of the suitable classes. The class hierarchy is shown in Figure 2. By default, OWL makes an open world assumption, which means that anything not mentioned in the Ontology is neither ‘false’ nor ‘impossible’, but rather ‘possible’. Thus, objects and truths that are ‘false’ or ‘impossible’ must be expressed explicitly as such in the Ontology. All classes at the same hierarchy level must thus be specified as Disjoint, which means that for any pair of classes that do not have an ‘isA’ subclass connection, an instance cannot belong to both classes. A Component Type cannot simultaneously be an ‘Annotation’ and a ‘Detection’, or a production line cannot be ‘AntennaManufacturing’ and ‘LiftManufacturing’.

Then, the Object Properties that represent the connections between the classes were defined. Table 1 provides a summary of Object Properties, including their domains (representing the ‘subject’ and answering the question ‘who?’) and ranges (representing the ‘object’ and answering the question ‘what?’). The majority of the Object Properties are declared as functional, which means that they link a single instance of the range class to only one specific instance of the domain class. For example, an ‘Operator’ is carrying out one ‘Task’ at a time, at one specific ‘ProductionLine’, wearing one ‘HUD’, so the Object Properties ‘performsTask’, ‘conistsOfTask’, and ‘hasHUD’ are functional, but a Production-Line can have many components, so the Object Property ‘incorporatesComponent’ is not functional. All Object Properties are disjoint, indicating that they are distinct properties reflecting distinct connections. Then, as in the case of Object Properties, the appropriate Data Properties were defined, which show associations with a Data Type (integer, boolean, etc.) rather than another class in their range.

Table 2 summarizes the Data Properties, as well as their domains and ranges. For each instance of the Domain Class, all Data Properties are functional and accept a single Data Type value. The supporting components were added as Ontology instances of the Component Class once the Ontology was built. This was achieved by creating a distinct Instance for each valid LoD for each Component Type.

#### 4.3.3. Priority Rules

The first set of rules were defined from the knowledge acquired during several co-creation workshops with the three use cases’ experts. Through the workshops, it was possible to select the Component Types and the components, suitable for each task in the different use cases. The defined priority rules are depicted in Box 1.

Box 1 **Priority rules template.**

Where for each *Production Line*, when the operator performsa Task and s/he is presented with the UI components that
take part in this *Task* their respective Component Type is given
a Priority value.

The ‘Pellet’ OWL 2 reasoner was utilized for the Ontology Reasoner, and a separate Python program was created using Owlready2 to infer the final active reactions (see Box 2):

Box 2 **SWRL inference rules for reactions.**

Operator(?o), Component(?c), performsTask(?o,**Task**),

consistOfTask(**ProductionLine**, ?t),

hasComponent(?u,?c), entailsComponent(**Task**, ?c),

employs(?c,**ComponentType**) →

hasPriority(**ComponentType**,**Priority**)

#### 4.3.4. Utility Rules

The rules for calculating the utility score of the components were developed on the basis of the priority of the Component Types and the sequence in which the LoDs were determined. The goal is to assign more weight to higher priority Component Types and lower-ranked LoDs. Specifically, a component’s utility score will increase if the priority of the Component Type it belongs to is high. In addition, the utility score is larger for components of a given Component Type if their LoD is lower in the ordering. Therefore, a component’s utility score is determined firstly by its Component Type; and secondly by its LoD, as components with a higher priority Component Type will always have a higher utility regardless of LoD. In order to implement all the above, the range for the component’s utility score is set to be a range [0.00, 1.00], with two decimal places. The first decimal place is determined by the priority of its Component Type, and the second by the LoD’s rank. To be more precise, the first decimal is determined by:

(1) 1−0.1∗Priority,

where Priority is a real number between 1 and 10, with 1 being the highest and 10 the lowest. Depending on how the LoDs and the component’s LoD are ranked, the second decimal point is set to either 0.09, 0.05, or 0.01. Specifically, 0.09 is assigned to the components with the most relevant LoD for the current context, which are first in the ordering, while 0.01 is assigned to the components with the least relevance and are thus last in the ordering. The pertinent inference rules for utility calculation are depicted in Box 3.

Box 3 **SWRL inference rules for utility.**

Operator(?u), Profile(?p), Component(?c), ComponentType(?cT), hasProfile(?u,?p), hasComponent(?u,?c), hasComponent-
Type(?c,?cT), hasExpertise(?p,**Expertise**), hasLoD(?c,**LoD**),
hasPriority(?cT,?r), multiply(?s1,-0.1,?r), add(?s2,1,?s1),
add(?s,**LoDUtility**,?s2) → hasUtility(?c,?s)

For each possible combination of Expertise and LoD, the LoDUtility, which is the appropriate value from the set 0.09, 0.05, is added to the priority relation described above to produce the utility.

#### 4.3.5. UI Optimizer

The UI Optimizer follows an approach similar to [11] for selecting the most appropriate component to display and for computing non-overlapping widget placements. For the microelectronics PCB Defect-Detection process, the operator observes the PCB on the AR glasses, and for every microchip, there is an annotation widget attached to it indicating the status of the chip. In the original approach, the annotation widget could be placed by the UI Optimizer above, left or right of the corresponding detection. These positions are extended with the bottom place, so that the annotations of the bottom row of microchips can be placed at the bottom side.

### 4.4. Rendering Framework

The Rendering Framework is responsible for the visualization of the information as defined by the Decision Maker.

#### 4.4.1. Deserializer

With the MQ Protocol, the Decision Maker Component communicates with the Rendering Framework component, sending the selected GUI widgets to be displayed by the UI Optimizer. The messages are transmitted in JSON format. However, in order for the information to be analyzed, processed, and visualized accordingly, the messages need to be deserialized in formats and data structures that are comprehensible from the Unity Engine and the C# scripts that form the logic of the application. The Deserializer is responsible for that and stores the information in a Queue structure.

#### 4.4.2. Renderables Queue

In a C# script attached to the Unity application, a Queue called ’Renderables Queue’ is initialized, which stores all the renderable elements that will be visualized in the Unity Scene. All of these components are Unity GameObjects, so the Queue is a GameObject as well. This way, there is no need for recreating GameObjects that have already been visualized previously.

#### 4.4.3. Tracking Dictionary

For each renderable element that is going to be visualized, it is necessary to keep track of it over the updated frames and be able to access it and trace it. It is required to know which elements are displayed in order to prevent visualizing the same element multiple times. Hence, a unique ID is assigned to each element and inserted in a C# Dictionary, where each unique ID is mapped with the specific element.

#### 4.4.4. Renderables Instantiation

When a GameObject is ready to be visualized, the Renderables Instantiation function is called up. This function spawns on the Unity Scene the GameObject on the position that is determined by the Decision Maker. Some of them present dynamic information, mostly with text and the color of the widget. At this point, the appropriate information for the widgets is assigned. Finally, the Instantiation function is called for every item in the Renderables Queue.

#### 4.4.5. Adaptive UI Widget Library

The adaptive UI widgets are polymorphic graphical user interface components designed to display specific information in the user’s field of view through AR glasses. They are a crucial part of the rendering and interaction framework since they show context-relevant information on the screen based on the pilot use case requirements. The widgets are self-contained AR modules that provide understandable information to enhance the operator’s SA. The widgets serve various roles, such as keeping the operator updated regarding manufacturing processes’ progress, and production quality. Each widget is developed using the Unity 3D game engine (version 2022.3.16f1) to ensure high-quality AR rendering. Unity is a powerful 2D and 3D programming engine favored for creating games, interactive movies, VR/AR experiences, and similar applications. It streamlines interactive system development with core concepts like GameObjects and Components. GameObjects are the foundation of Unity projects, containing code for object features, while Components, implemented as C# scripts, enhance functionality by adjusting attributes and behavior for desired outcomes such as animation and interaction. Therefore, the system’s rendering and interaction framework renders each widget as a prefab GameObject in Unity, with its own rendering and interaction capability. As such, the widgets are modifiable GUI software components using the Unity Canvas UI framework that provides general GameObject functionality.

#### 4.4.6. Widget Design

The system’s adaptive UI widgets have been designed so that can be rendered in different LoDs for visualizing a piece of information. The widgets’ design was assessed and fine-tuned by multiple workshops with different stakeholders from the three studied plants to determine the requirements for the components and the pertinent information to be delivered. The initial designs were implemented by overlaying the widgets onto realistic background scenes of the shop floor of each studied site, whereas the final designs were assessed by three user experience experts, as interactive mock-ups displayed on AR headsets estimating the usability of each component in shop floor contexts and activities. The designs were modified to address the usability issues that emerged from the expert base evaluation. The final set of widgets is described next. All the widgets are classified into different categories based on the type of data and content they visualize as well as the purpose they serve: Defect Detection widgets; Defect Cause Information widgets; Menu; Warnings; Suggestions; and Shop floor process progress.

#### 4.4.7. Widget Prototypes

In this section, for each widget category, a list of the available widgets is provided, with a detailed explanation of their characteristics, most suitable use cases, and a comprehensive summary table of all library widgets.

**Figure 3:**
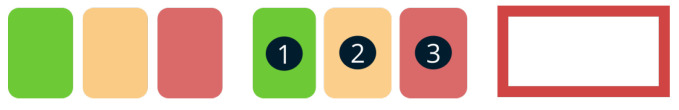
Widget for annotating PCBs. Numbers help the operator correlate alerts with specific defective areas.

Defect Detection. This category includes all adaptive UI widgets that digitally annotate a defective product within the user’s field of view. These widgets overlay the detected item with a colored shape, which may be an outline or a colored box, depending on the widget’s LoD. The color used indicates the product’s status, i.e., green signifies that the product is not defective, yellow indicates uncertainty about its quality, and red denotes that the product is defective and should be rejected. In case multiple areas need to be annotated, each annotation is numbered so that the operator can easily corellate pertinent alerts they receive in lateral components with each different area. Figure 3 depicts all the different representations of a defective PCB for the microelectronics case study.

Defect Cause Information: For every detected defect, annotation widgets provide coherent information elucidating the reason for the observed defect. The annotation widgets support two LoDs. The Low LoD version of the widget consists of an icon indicating the problem while the High LoD version consists of the same icon and a short explanation. In many cases, the High LoD widget also includes graphical representations and charts of measures provided by the system providing useful insights into the operators regarding the defect’s root cause. Additionally, specific widgets are used to denote the acceptability of a product by superimposing colored dots onto it, in Low LoD, as per the following color code: green for acceptable, yellow for borderline acceptable, and red for rejected. In High LoD, an icon is used instead of a dot, following the same color code.

Menu: The menu is an important widget that needs to be present when the system provides various functionalities. A menu widget is usually placed at the top side of the AR headset display and can follow a horizontal or vertical orientation. The Low LoD version of the menu component consists of a set of icons only, while the High LoD version includes the same icons accompanied by a text label describing a specific task (e.g., product inspection).

Warnings and Suggestions: Another type of widget that is employed in the system is the Alert and Suggestion. This widget is a box where notification tabs instantiate whenever there is a warning for the operator regarding a defect or upcoming issues and suggestions for handling these issues.

Shop Floor Processes Progress: Some widgets support the execution and completion of the system’s various processes. The first type of widget in this category is pop up messages that prompt the user to initiate a process or move to the next step. These widgets need to grab the user’s attention so they are usually placed in the middle of the screen. Conversely, for occasions that the user should continuously monitor information related to an ongoing process, pertinent widgets are placed at the bottom right corner of the headsets’ display so as not to distract them from their main task, and look at them only when needed. These widgets provide suggestions and solutions for resolving issues that have been detected, preferably in High LoD when the context of use permits it. In several cases, results and insights that emerge from the analysis of the production line might need to be studied by the operators on the shop floor, so as to better understand subtle production issues that might lead to potential defects. To address this need, chart and graph diagram widgets are shown on their headsets relatively placed in the area of the production line so that the operators are able to correlate the received information with the production and take pertinent action if needed.

### 4.5. Renderables and Natural Interaction Processing

Each gesture performed by the operator during the application’s use may cause a reaction if the right conditions are met. A reaction is an action or behavior that the system performs in response to the user’s gestures. The present component is responsible for processing the user’s gestures and determining whether and which reaction is triggered. This component engages in a reasoning process based on an ontological model to make these determinations. To better comprehend the Ontology-based reasoning for inferring the reactions, it is first necessary to explicate the taxonomy derived from [74]. This taxonomy was utilized in the creation of the gesture vocabulary for the system. The vocabulary is then explained with each selected gesture and the corresponding reactions. Finally, after all the necessary parameters have been examined, the reasoning process involving the Semantic Web Rule Language (SWRL) rules is expounded upon.

#### 4.5.1. Context-Driven Adaptive Interaction Ontology-Based Reasoning

As already mentioned, the operator can perform a set of gestures to trigger various functionalities. When a user interacts with a system or device, such as issuing a command through a gesture or button press, it is important for the system to provide some form of response to indicate that the input has been recognized and understood. However, in cases where the user’s input is accidental or unintended, or if they are doing something else entirely, it may not be necessary or appropriate to provide a response. This is because responding to every input, even those that are unintended or irrelevant, can lead to what is known as the ‘Midas touch problem’ as [85] described. For example, if the system detects a gesture while the user is using their hands for another purpose beyond interaction with the system, and it responds with an error message or other feedback, this can be disruptive and annoying. To avoid the Midas touch problem, it is important for systems to be designed to recognize when a user’s input is intentional or accidental, and to provide appropriate feedback only in the former case. In order for a gesture to initiate a process, there are some conditions that need to be met, according to the Ontology model in Figure A1. More specifically, depending on the production line the operator is currently occupied in, as well as the task and UI components that are projected to them in the AR glasses (modeled in the Ontology), the Ontology Reasoner determines the reaction that is triggered. This mechanism also helps distinguish the different reactions that the same gesture may correspond to. For example, thumbs-up triggers the start of the inspection process and the play of a video, but the inspection should only begin if the pertinent pop up message is displayed, the user is in a production line that supports the inspection process (e.g., microelectronics production line) and performs a defect-detection task. In a nutshell, through Ontology-based reasoning and context-awareness, the current work addresses the ‘Midas touch’ problem and ensures the punctuality of system responses. For the implementation of this model, we had to enrich the Ontology that was previously developed in the Protégé tool. More specifically, a boolean flag is set to true when all the necessary conditions are met for a reaction to be triggered; and false if they are not. For example, if the operator is working in the microelectronics assembly line, monitoring the production process, the UI component that is displayed in the HUD is the microelectronics_OutputInspection and if the operator performs a ThumbsUp hand gesture, the StartOutputInspection process is activated. In contrast, if each one of the conditions is not satisfied and the operator performs a gesture, no reaction should be activated. To determine which reaction is triggered and when, a set of SWRL rules needed to be specified. This set of rules assigns a Boolean value to the reactions based on whether they are activated. In the process of defining the SWRL rules, we had to specify the Tasks that are involved in each Production Line, and the components that support gesture interactions and are part of the specified tasks. Combining these four aforementioned parameters, we distinguished the reaction that is triggered with every combination when a specific gesture is performed by the PLO. Based on the Ontology model and the approach described above, the rule template depicted in Box 4 was created.

Box 4 **Template of reactions triggered by a gesture realization for a specific task.**

Where for each *Production Line*, when the operator performsa Task and she/he is presented with the UI components
that take part in this *Task* and she/he performs a gesture,

if a reaction is provided by the *UI components*

in the AR glasses, the gesture will trigger this *Reaction*.

The ‘Pellet’ OWL 2 reasoner was utilized for the Ontology Reasoner, and a separate Python program was created using Owlready2 to infer the final active reactions (see Box 5):

Box 5 **SWRL inference rules for the final active reactions.**

Operator(?o) ∧ Component(?c) ∧ performsInteraction(?o,Interaction) ∧ supportsInteraction(?c, Interaction) ∧triggersReactionInteraction, Reaction) ∧entailsComponentTask, ?c) ∧ employes(?c,ComponentType) ∧ consistOfTaskProductionLine, Task) ∧providesReactionComponentType, Reaction) ∧
isActiveReaction, true)

#### 4.5.2. Gesture Vocabulary

Stepping on the core findings that were reached from the state-of-the-art review on gesture-based interaction in AR applications in different contexts of use, a gesture vocabulary for the system environment has been designed. For the vocabulary design, we also considered the tasks that the PLO has to complete in the different production lines of the three pilot sites, and the reactions they require. It is a key issue that the PLO’s focus is primarily on his environment, while being assisted by the AR technology. For that matter, the proposed gestures are supposed to not require the operator’s full attention in order to be executed.

Finger Count: Through the systematic literature review, we noticed that a common way for selecting an item from a menu is by finger count. More specifically, the users lift as many fingers as the index of the menu item they wish to select (e.g., if they want to choose the second element of the menu, they need to lift two fingers). The menu component in the operator’s field of view is designed to include three items; thus, by integrating the finger count gesture, the operator has to use only one of his hands. The finger count gesture is also natural to the users, since it is used in everyday life as well.

Opening and Closing Fist: Through the various tasks the PLOs have to complete, whenever a defect occurs in the production line, they might need to stop the production for a while to avoid upcoming defects and recalibrate the systems involved. Since this operation requires a rapid reaction, the gesture of closing and opening the fist is suitable for this interaction.

Thumbs-Up and Thumbs-Down: Through the AR UI, the PLOs can initiate specific processes (e.g., Output Inspection, Tools Checking, etc.), by selecting a menu option with the finger count gesture. In order to initiate one of these processes, after a pop up message appears, the PLO has to confirm by performing the thumbs-up gesture, or decline to start the process by performing the thumbs-down gesture.

Swipe Up and Swipe Down: The swipe gesture is used for the recalibration of the system. For example, if the glue quantity is found excessive after the glue-dispensing process, the PLO will have to decrease the nozzle pressure. This can be done by a swiping down gesture, simulating the movement of lowering a bar. If the operator needs to increase the value of a parameter in the system, she/he can perform the opposite gesture (swipe up).

Slide Left and Slide Right: The slide gesture is used for the calibration of the motors that need to be turned right or left.

Wave: The PLO can wake up the system when they need it, by waving their hand in the air.

Confirmation: The operator performs the confirmation gesture to signal the completion of a subtask. For instance, when the operator adjusts the valves for elevator speed, she/he performs the confirmation gesture, indicating the completion of calibration.

### 4.6. Application Description

The following subsections present three prototype applications developed by employing the proposed library, considering the requirements specified for each studied factory. The three applications are tailored for each production line, employing specific features and functionalities that meet the operators’ needs. Through these applications, we could assess the feasibility of the discussed library in real-world scenarios and evaluate its potential to foster zero-defect productions.

Microelectronics Production Use Case. The application designed for the microelectronics production line starts with the Home Screen. In the Home Screen there is the menu in the top left corner of the screen that includes the options Glue Dispensing and Inspection. The user can choose between two options by using hand gestures, where the finger count corresponds to the respective item (Finger Count One for Glue Dispensing and Finger Count Two for Inspection).

The primary feature of this application is the Inspection mode. In this mode, users can examine a PCB board to identify any defective parts that should be rejected. Upon selecting the Inspection mode, a pop up message appears, instructing the user to position the PCB in front of them and initiate the process. Once prepared, users can commence the Inspection by making a thumbs-up gesture.

**Figure 4:**
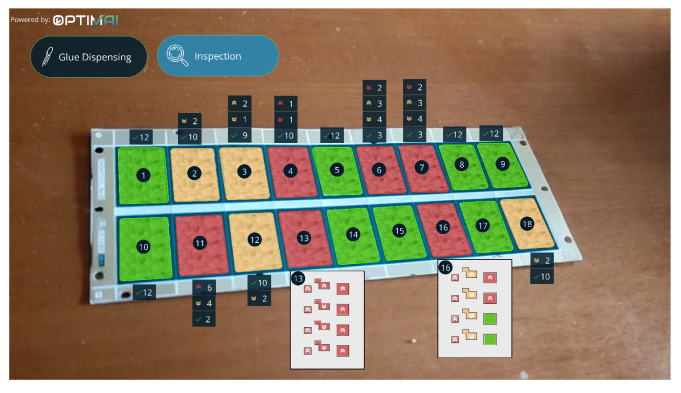
Application UI during the inspection mode of the PCB. Colored squares highlight modules and are numbered for reference.

During the Inspection process, users view the PCB through the AR glasses, while on the UI, the PCB modules are highlighted with annotated colored squares and corresponding numbers, as shown in Figure 4. The color of each square signifies the module’s status: green for acceptable, yellow for borderline acceptable, and red for rejectable. Each module is accompanied by an annotation widget. Some widgets are Low LoD, visible on the left modules, while others are High LoD, such as modules 13 and 16. The Low LoD widgets provide information on the number of glues in the module, indicating whether they are excessive and rejectable, insufficient and rejectable, excessive and borderline acceptable, insufficient and borderline acceptable, or within the normal range. On the other hand, the High LoD widgets offer a detailed representation of the inside of the module. Each glue area is annotated with its status color and an arrow indicating the sufficiency of the glue.

In an alternative inspection mode, the PCB is examined after the routing process is finished. Areas of interest are identified and color-coded with three status colors (green, yellow, red). Each colored area is accompanied by a widget that specifies the nature of the defect. Potential defects include an oversized chip, undersized chip, or a damaged chip.

In the last inspection phase, the wafer is scrutinized after the sawing process, revealing faulty areas on the wafer. A corresponding message elucidates the nature of the defect. Detected defects may include chipping, cracking, or incorrect depth.

Antenna Production Use Case. The application designed for the antenna-production line starts with the initial Home Screen that contains only a menu. The menu includes the Watch Replay, Inspection, and Defect Alerts options. Users can navigate through these options by performing corresponding finger counts—one for Watch Replay, two for Inspection, and three for Defect Alerts. Opting for the Defect Alerts option activates the real-time notifications box positioned at the bottom right corner of the user interface. In this notification box, alerts for each inspected antenna are displayed, presenting crucial information such as the timestamp and the status of the antenna. When a defective antenna is identified or the system anticipates a potential defect, a pop up message appears in the PLO’s field of view. In the case of a predicted defect, the message also indicates the reasons for generating this prediction. The operator can then stop the production with the closed fist gesture and watch a replay video of the occurred defect or a simulation video of the predicted defect. Video replay control is facilitated through mid-air gestures displayed at the bottom of the screen.

For cases where a defect is already detected, the operator can choose the ’Inspection’ menu option. Placing the antenna in front of them and initiating the inspection with a thumbs-up gesture; the system proceeds to identify and annotate the defective areas using a highlighter frame. Additionally, it provides information about the specific cause alongside the defected area through relevant icons.

Lift Manufacturing Use Case. The application designed for the lift manufacturing use case encompasses diverse scenarios to cater to the multiple steps involved in the manufacturing process. The Home Screen features a menu situated at the top left corner, comprising three options: Check Order, Manual Setup, and Unit Testing. Additionally, the top left corner displays the order ID currently being processed.

To check an order, the user initiates the process by performing the Finger Count One gesture. Subsequently, the tools and unit parts undergo examination. The user triggers the process with a thumbs-up gesture, initiating the tool-checking process. After the tool-checking process concludes, the system transitions to the unit part-checking process. Upon completing the comprehensive checks, the system prompts the user to commence the manual setup. During this phase, the user is required to adjust the valve blocks while simultaneously monitoring the velocity, sound and pressure values.

Finally, the unit-testing process begins and the operator can see all the diagrams in their field of view in order to inspect them. Following the inspection, the operator is presented with three options: to print the results, retest in case any issues were identified, or conclude all processes.

## 5. Evaluation

In this section, the evaluations conducted on the developed applications utilizing the proposed extended library of GUI widgets will be discussed. A cognitive walkthrough involving 10 experts and three user-based evaluations were carried out at three pilot sites, involving a total of 30 users (this study was approved by the Ethics Committee of CERTH, Greece (REF No: ETH.COM.75)). For each evaluation, the methodology and results are analyzed to identify specific issues per use case. The section concludes with a discussion driven by the aggregation of results across pilot sites.

### 5.1. Cognitive Walkthrough

#### 5.1.1. Method and Procedure

Cognitive walkthrough is an expert-based usability inspection method, whereby the evaluator uses the system to perform tasks that a typical user of the system would need to accomplish and evaluates the interface by responding to specific questions, with the goal of identifying aspects of the interface that could be challenging to new users [82]. The questions that the evaluator examines during their walkthrough to the system are the following:

- (Q1) Will users try to achieve the right result? In other words, do users understand that the action (step) at hand is needed to reach their larger goal?
- (Q2) Will users notice that the correct action is available? In other words, is the interactive element that achieves the step visible or easily findable?
- (Q3) Will users associate the correct action with the result they are trying to achieve? Perhaps the right button is visible, but will users understand the label and will they know to engage with it?
- (Q4) After the action is performed, will users see that progress is made toward the goal? Based on what occurs after the action is taken, will users know that this action was correct and helped them make progress toward their larger goal?

In the context of the current work, the cognitive walkthrough method was applied to assess the prototype developed for the antenna-production line, aiming to elicit potential areas of improvement not only for the specific case study but in general for the GUI widgets library which would be extended to the implementation of the remaining use cases.

The evaluation took place in an online workshop format where a total of 10 UX and domain experts participated, namely UX designers, developers, as well as engineers, operators, and managers from the antenna factory. To familiarize the participants with the system, they were initially shown a video of the system demonstrating how it is expected to operate. Specifically, two scenarios were demonstrated and then evaluated: (i) the detection of a defect by the system, and (ii) the prediction of an upcoming defect by the system. Then, participants were introduced to the cognitive walkthrough method and were explained the procedure that would be adopted. The discussion that followed was coordinated by the workshop facilitator, going through all the individual UI screens and asked participants to respond individually to each of the cognitive walkthrough questions. Aiming to support quantitative analysis of results, besides the qualitative feedback that was expected to be collected, participants were asked to indicate their opinion on the above questions by treating each question as a likert-scale and providing their rating on a 5-point scale.

#### 5.1.2. Results

Scenario 1—Defect Detection. In this scenario, the operator is notified through their AR glasses that a defect has been detected at the production line. The user has activated the defect alerts log and since a defect has been detected, a pop up message is displayed in their field of view. Following defect detection, according to the scenario, the user stops the production by performing the gesture of a closing fist. Then, aiming to acquire insights into the detected defect, the user selects watching the video replay of the defect, being able to control the replay through four distinct gestures. After watching the video, the operator decides to do a manual inspection of the defective antenna and initiates the process with a thumbs-up gesture. Last, they view the inspection results overlaid on top of the actual antenna. Table 3 summarizes results from the cognitive walkthrough performed on the first scenario, displaying the average score (M) and standard deviation (SD) for each of the questions (Q1–Q4) introduced in Section 5.1.1.

It is evident that all questions were highly rated and participants’ opinions were generally aligned across all aspects examined. Further discussion with the participants on each screen highlighted the following issues:

- Overall, the system was found to be quite simple and straightforward for the users.
- A general concern highlighted was that the AR glasses should be comfortable for the operators to wear during their working hours and reliable (e.g., sufficient battery) supporting them through their daily tasks.
- Regarding defect detection, the evaluators were skeptic about the usefulness of the replay and inspection functions for highly experienced operators. Nevertheless, these functionalities were appraised for operators under training, as well as for novice operators. The stop production function will be useful for all types of users.
- The video replay functionality was characterized as very helpful since the operator cannot see inside the machine.
- For the inspection functionality, the evaluators expressed concerns about the system’s ability and accuracy concerning detecting all the defects. In fact, they pointed out that some defects are usually detected with ample light shed on the antenna.
- It was proposed that the system should be trained by the operators by confirming or rejecting a defect detection.
- Furthermore, it was pointed out that the definition of defect is needed during training and for inexperienced operators, while for skilled operators it will be enough to highlight the position of the defect on the antenna.
- The gestures were found to be simple and intuitive. Also, the fact that there are visual hints everywhere was praised and found helpful. However, there was a concern expressed concerning how easily gestures would be detected in a visually noisy background such as that of a factory.

Scenario 2—Defect Prediction. In the next scenario, the system predicts a defect so it alerts the user with a pop up message. Again, the user can stop the production and watch the simulation video of the upcoming defect.

Table 4 presents the results from participants’ responses for the four questions (Section 5.1.1) regarding each screen evaluated in the second scenario.

For this scenario as well, ratings to questions of the cognitive walkthrough were rather high, meaning that the system is easy to learn and no major obstacles are expected to be faced by users. During further discussion with participants and elaboration on their ratings, the following remarks came about:

- Overall, it was highlighted that the User Interface is clear and intuitive and that it successfully guides the user throughout the interaction.
- Regarding defect prediction, it was pointed out that such a message would probably follow a defect-detection message, taking into account that defects are predicted after a number of defects occurring in a row. Therefore, it was suggested that the prediction message can be merged with the defect-detection message to avoid multiple pop ups, which would result in overloading the operator. In this process, the input from the scanner must be considered as well. In the explanation of the predicted defect, some information must be listed such as the kind of defect, the number of defective products, and the respective percentages. The operator should have the facts available to decide if they will stop the production. Then, the engineers can take actions in order to fix the production line as soon as possible.
- Furthermore, it was highlighted that the simulation video will not be so helpful for an operator, but it will be for engineers. However, engineers are expected to view analytics and any simulations on a PC and not in real time through AR glasses; therefore, this functionality could be omitted from the revised AR glasses prototypes

Discussion. The results of the evaluation were very positive regarding the usability and learnability of the system, highlighting that it is simple and clear, gestures are intuitive, and guidance is provided throughout the interaction. In addition, further discussions with the field experts yielded valuable findings for improving the specific AR glasses prototype. Specifically, the following changes were deemed necessary to be introduced:

- Defect-prediction messages should be merged with defect-detection ones, to avoid overwhelming the operator.
- Defect-prediction messages should provide details on the system’s rationale behind this prediction, such as the kind of defect anticipated, the number of defective products so far and the respective percentages.
- The defect-simulation video should be removed, as it will not be of use to the operators.

Findings from this early inspection that are generalizable and apply to the proposed widget library are as follows:

- When the system displays information on predictions, it should also make sure to provide an account of the underlying rationale to allow operators to better understand the basis of predictions and eventually trust the system.
- Defect-detection messages should be enhanced with defect-prediction information when available.

Other general findings regarding AR gesture-based systems are:

- When AR glasses are expected to be worn for long time periods (e.g., during working hours), comfort is a major concern, as well as reliability.
- When a specific gesture is expected by the users, a visual hint provided by the system acts as a safety net and supports the learnability of the system.

Regarding intelligent systems that are expected to be used in industrial environments:

- When automated decision-making is involved, a training by the PLOs would be beneficial to fine tune the system to the needs and requirements of each industrial set up and the procedures followed.
- Accuracy of predictions and detections is of paramount importance to the overall usefulness and acceptability of the system by its end-users.
- Accurate recognition of gestures in a visually noisy background may be challenging, yet it is a prerequisite for the smooth operation of the system and the support of operators.

Additionally, it turned out that in several cases it was believed that expert operators will mostly rely on their own expertise, rather than the system detections, while concerns were raised regarding the accuracy of detections and predictions. Future pilot evaluations should further focus on the issue of acceptability of the proposed technology and trust toward it. In any case, it must be clarified that the system is not expected to substitute human field expertise; instead, it aims to complement it by facilitating operators in quickly acquiring information that they may need to perform their daily job tasks. Finally, the pilot evaluation highlighted the potential of the system as a training tool and the advantages it may offer to inexperienced operators. Future work will aim to acquire additional insights on these aspects, comparing for instance user acceptability, perceived ease of use and perceived usefulness between novice and expert users.

### 5.2. User-Based Evaluation

#### 5.2.1. Method and Procedure

User-based evaluation is the process through which the users of a product, service, or system evaluate its performance or efficacy. This sort of review often entails collecting input from users about their experiences with the product, service, or system, then using these data to find areas for improvement and make any required adjustments. The purpose of user evaluation is to verify that the reviewed product, service, or system meets the demands of its users and provides an effective and satisfying user experience [86].

The user-based evaluation aimed at responding to the following research questions:

- (Q1) Is the system easy to use?
- (Q2) Can the system support operators effectively and efficiently in their daily job tasks?
- (Q3) Are users satisfied with the system?

To this end, the evaluation was organized as a scenario-based evaluation combining user observation, standardized questionnaires, and interviews with participants. More specifically, through user observation the facilitator kept notes regarding task success, any errors carried out, help requests, and user remarks. Users were also asked to apply the think aloud protocol [87], that is to say out loud what they were thinking during the evaluation process. Two standardized questionnaires were employed, namely the Single Ease Question [88], which is used for assessing user satisfaction after each scenario, and the SUS questionnaire [89], which is used for assessing the usability of a system. Finally, a semi-structured interview was carried out to further elaborate on system aspects that users liked and disliked, and elicit suggestions for future system improvement. In terms of procedure, the whole evaluation process took place at the shop floor of each pilot site. For the setup, a laptop was used, the AR glasses, as well as a defective product and the Bill of Materials. Participants were welcomed and explained the aims and objectives of the test, the procedures followed for the protection of their data, and that they could revoke their participation and consent at any time without any repercussions. Any questions were clarified and then participants were given an informed consent form to sign. Overall, each user-testing session was structured as follows:

1. Welcome and introduction to the test;
2. Demonstration of the system and short training;
3. Filling-in of a background information questionnaire, asking about gender, age range, professional expertise, and previous experience with AR systems;
4. Execution of task scenarios, with participants filling in the SEQ questionnaire after each task;
5. Filling-in of the SUS questionnaire;
6. Interview and debriefing.

Before the session, the interaction with the system was explained to participants, detailing all the possible gestures and their operation. More specifically, participants were guided through the system via a short tutorial performed by the facilitator. For each session, the facilitator would wear the AR glasses, while the displayed contents of the glasses were also projected on the laptop screen. The facilitator then performed a detailed walkthrough of the system with the appropriate gestures for the interaction. When the training was completed, the facilitator answered users’ questions if there were any. Furthermore, as all participants were first-time users, they were also provided with a printed summary of the gestures, and were instructed to use it whenever needed during the experiment. In addition, participants were informed that they could do the experiment sitting on a stool or standing up. Meanwhile the facilitator would be monitoring the whole process through the laptop’s screen while also taking handwritten notes of user comments, interactions with the system, errors, and assistance required, as well as task success. This evaluation aimed at determining the usability of the developed User Interface by users who are specialists in the field and who perform the inspection process in the context of their daily job tasks. Furthermore, the intuitiveness and naturalness of the proposed gestures was evaluated.

##### Participants

A total of 30 users participated in the evaluation, 3 females and 27 males, all employees of the three factories of the pilot sites. The age of the participants varied from 18 to 64 years old, where most users under the age of 35 years old had less than 5 years of expertise and the ones older than 35 had more than 10 years of expertise. While our initial intention was to involve PLOs exclusively, since they represent the primary target users of the system, this was not fully feasible due to scheduling constraints and the limited number of available PLOs at each pilot site. To address this, we broadened the participant pool to include other relevant factory roles, such as directors, engineers, technicians, and managers. These individuals were selected based on their familiarity with the production environment. Finally, 76% of the users had previous experience with computer applications but only 6% had experience with AR applications.

##### Scenarios

Defect Detection in the microchip-production line. In this user evaluation, the gesture-based AR application for the PLOs of the microelectronics-assembly production lines was evaluated. More specifically, the application displays the UI that a user has to interact with, through hand gestures, in order to perform the PCB defect-inspection process. The system was evaluated through three main tasks.

Task 1: Initiate the inspection process. In this task, users were asked to initiate the PCB inspection process. In the initial screen of the application, there is a menu with three options. In order to initiate the PCB inspection process, the user has to select the second menu item with the finger-count gesture. The task is successful when the user performs the correct gesture and the pop up message shows up on the screen. Then, the user has to carry out a thumbs-up gesture to start the inspection.

Task 2: View inspection analysis results. Users were asked to view the inspection results and report which microchips were defective and why, which were good, and which acceptable. This task is considered successful if the user is able to see the defects and point out the defective, good, and acceptable microchips and provide a rationale for this.

Task 3: Deactivate the system. Finally, the user was asked to deactivate the system by performing the correct gesture.

Defect Detection in the antenna-production line. In this user evaluation, the gesture-based AR application for the PLOs of the antenna-production line was evaluated. In this case, the application displays the UI that a user has to interact with, through hand gestures, in order to perform the antenna defect-inspection process and view prediction analysis results. The system was evaluated through two main tasks.

Task 1a: Enable defect updates. In the initial screen of the application, there is a menu with two options. In order to initiate the defect updates, the user has to select the second menu item with the finger-count gesture. The task is successful when the user performs the correct gesture and the pop up message with the alerts shows up on the screen.

Task 1b: Stop Production. The alerts that are received regarding each antenna passing through the machine indicate that the antennas are ok. After a few seconds, the system detects a defective antenna, notifies the user, and prompts them to stop the production with the close fist gesture. The task is considered successful when the user performs the gesture and the pop up message for initiating manual inspection appears.

Task 1c: Carry out defect inspection. The user has to perform the gesture finger count one to select the first item from the menu as the pop up message suggests to begin the inspection process. Then, they have to position the antenna in front of the glasses and perform the thumbs-up gesture. The task is considered successful when the user can see the red rectangle surrounding the defect on the antenna and repeat the message displayed for the type of defect.

Task 1d: Return to Home Screen. Finally, when the inspection is completed, the user is prompted to go to the Home Screen with the correct gesture to start the second scenario.

Task 2a: Select the appropriate menu item for obtaining updates. The user has to initiate the defect-update process again by selecting the second menu item with the finger-count gesture.

Task 2b: Prediction analysis. This time, the system predicts that there is an upcoming defect. Therefore, the system prompts the user to stop the production and watch the simulation video of the defect by selecting the third menu item. The task is complete when the user has watched the video.

Task 2c: Deactivate the system. Finally, the user has to deactivate the system with the closed fist gesture.

Bill of Materials inspection in lift manufacturing. In this user evaluation, the gesture-based AR application for the PLOs of the lift manufacturing was evaluated. In this case, the application displays the UI that a user has to interact with, through hand gestures, in order to perform the lift Bill of Materials inspection process, calibrate the motors for the lift velocity, and view unit-testing diagrams for sound, velocity, and pressure. The system was evaluated through three main tasks.

hlTask 1a: Initiate the inspection process. The user has to perform the gesture finger count one to select the first item from the menu. Following the prompt from a pop up message, the user then proceeds to start the inspection process by making a thumbs-up gesture.

Task 1b: Tool collection for the inspection process. The user has to directly look at the tools and gather the ones that are highlighted by the system. The task is considered successful if the user picks up all the tools indicated by the system.

Task 1c: Lift Bill of Materials inspection. In this task, the user must focus directly on the lift Bill of Materials and verify each part against the type specified in the order. If a part matches the indicated type, the user should perform a thumbs-up gesture. Conversely, if a part does not match the specified type, the user should perform a thumbs-down gesture and then proceed to replace the part with the correct type. This process continues until all parts listed in the Bill of Materials are correct according to the specified types.

Lift velocity calibration. Task 2a: Initiate the calibration process. The user has to perform the gesture finger count two to select the second item from the menu. Following the prompt from a pop up message, the user confirms the initiation of the calibration process by making a thumbs-up gesture.

Task 2b: Calibration of the lift velocity. The user uses gestures to calibrate the velocity of the lift. The calibration is conducted by turning each of the three motors to the right or left, depending on the required adjustment. The user selects the appropriate motor performing with the finger one, two, or three gestures, respectively. Then using the slide left or right gesture, they initiate the turn of the motor. To stop the movement of the motor, the operator performs the closing fist gesture.

Task 2c: Complete the calibration process. The user has to perform the confirmation gesture to finish the calibration process.

Lift unit testing. Task 3a: Initiate the unit-testing process. The user has to perform the gesture finger count three to select the third item from the menu. Following the prompt from a pop up message, the user confirms the initiation of the unit-testing process by making a thumbs-up gesture.

Task 3b: Lift unit-testing diagrams. The user carefully looks at the diagrams in front of them. The task is considered successful if the user indicates the correct max value of the sound in those diagrams.

Task 3c: Complete the unit testing. The user has to close the diagrams and complete the step using a thumbs-up gesture.

##### Results

This section presents the key findings from the evaluations. Initially, we discuss the success of each task based on experiments conducted at the three pilot sites. Subsequently, participant satisfaction regarding task performance is examined through the analysis of responses to the SEQ. Insights into system usability, as perceived by participants, are derived from the results of the SUS questionnaires. Qualitative feedback from participants, obtained through semi-structured debriefing interviews, is also summarized. Finally, this section provides valuable design guidelines for gesture-based AR systems in industrial environments.

Task Success. Regarding the microelectronics application evaluation, all participants successfully completed task 3 by performing the correct gesture. For task 1, 70% of the participants performed the correct gesture. The rest of the participants were somewhat confused and performed the wrong gesture with 2 of them performing the correct one after not seeing a response from the system (therefore, the task was marked as Partial Success), and the other receiving a hint from the expert (hence, the task was marked as failed). For the second task, 90% of the users could distinguish the acceptable and borderline acceptable microchips. However, some of them (56%) could not explain the reason behind the defective microchips, relating the defectiveness with the glue annotation areas and numbers marked as partially successful. Furthermore, one of the users was colorblind and could not identify the difference between the colored microchips; thus, they inferred the answer, and the task was marked as ‘Fail’. In summary, Task 3, was 100% successful, while Task 1 was 70% successful with 20% partially successful and 10% failed, and Task 2 was 40% successful, 50% partially successful, and 10% failed.

During the antenna-application evaluation, 100% of the participants successfully completed Task 1b, 2a, and 2b. Regarding Task 1a, 20% of the participants were not sure how to select the menu item and had to consult the cheatsheet to perform the correct gesture. Regarding Task 1c, during the inspection process, the users were asked about the type of defect and three of them could not determine the type of defect. The reasons behind this were that the font size was considered somewhat small and also that the message was not clearly seen due to misplacement of the glasses. In Task 1d, 20% of the participants did not remember the gesture for going back to the Home Screen so they needed to look at the cheatsheet. Finally, for Task 2c, 20% of the users also did not remember how to deactivate the system and they performed the correct gesture after consulting the cheatsheet. None of the tasks were completely failed. Task 1b, 2a, and 2b were 100% successful. Task 1a, 1d, and 2c were 80% successful and 20% partially successful, while Task 1c was 70% successful and 30% partially successful.

During the evaluation of lift manufacturing, all participants successfully completed the tasks related to the selection of menu items (Tasks 1a, 2a, 3a), as well as the main tasks including the calibration of lift velocity (Tasks 2b, 2c), understanding unit-testing diagrams (Task 3b), and inspection of the Bill of Materials (Task 1c). However, during Task 1c, two participants reported difficulties in reading the text and understanding the detection indicators. Regarding the collection of the tools for the inspection process (Task 1b) 60% of the participants successfully completed the task, 30% partially successful, and 10% failed. The main reason for the failures, based on the observer notes, was the placement of the UI that indicated the tools to be collected, their quantity, and remaining items. Placed in the bottom right corner of the AR glasses, this UI was blurry for some participants, impeding readability. Moreover, one participant who failed the task was not familiar with English, which is the language of all the UIs. Furthermore, Task 3c, involving unit-testing completion, proved challenging for 20% of participants, who partially completed the task by performing the wrong gesture initially (Confirmation); however, they performed the correct one after the system did not respond. In summary, Tasks 1a, 1c, 2a, 2b, 2c, 3a, and 3b were successful, Task 1b was 60% successful, 30% partially successful, 10% failed, and Task 3c was 20% partially successful.

Overall, the most important difficulties encountered pertained to the recollection of appropriate gestures to perform and understand the icons for annotating the detected defects and parts of the Bill of Materials. This highlights the need for training users before the actual system usage, something which is expected to be the norm for the actual deployment of the system in the foreseen setting.

Satisfaction per task. In this section, we discuss the satisfaction of the participants regarding each task, based on their responses to the SEQ questionnaire. For each pilot site, the participants were asked to rate the difficulty of each task on a scale from 1 (= very difficult) to 7 (= very easy). The results are presented in detail in Table 5, Table 6 and Table 7.

**Figure 5:**
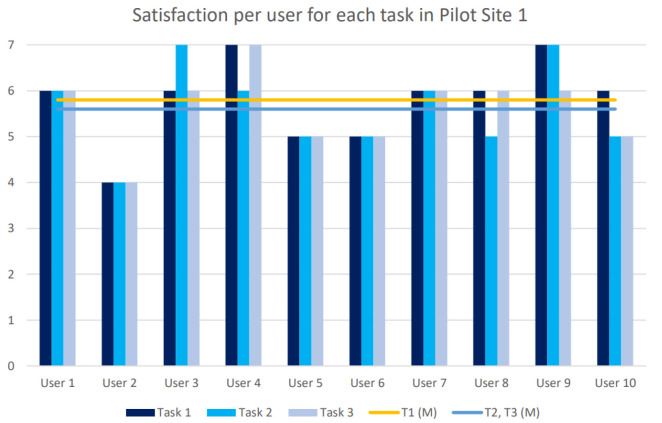
Diagram of satisfaction per user for each task for microelectronics application.

**Figure 6:**
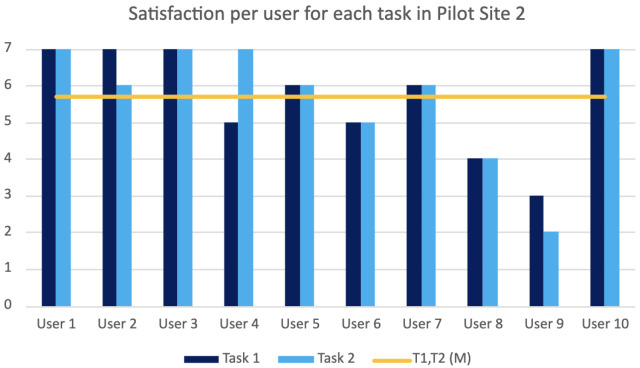
Diagram of satisfaction per user for each task for antenna application.

**Figure 7:**
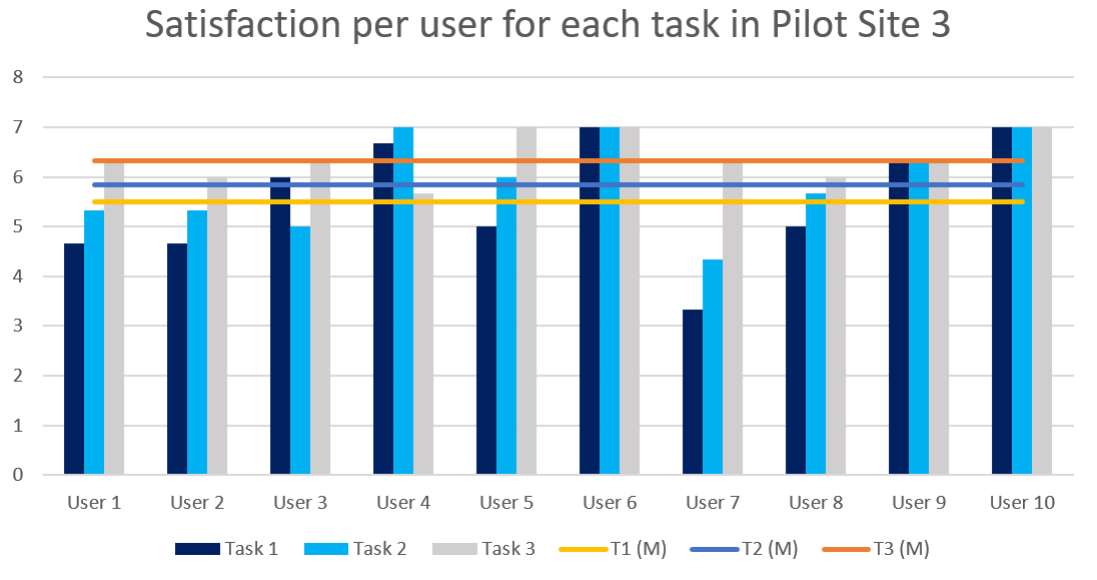
Diagram of satisfaction per user for each task for lift-manufacturing application.

For Pilot Site 1, as illustrated also in Figure 5, participants rated all three tasks as relatively easy, with average scores above 5.5, indicating good perceived usability. Task 1 was perceived as slightly easier than Tasks 2 and 3, though the differences are minor. The standard deviations are below 1.0, suggesting consistent responses across participants. For Pilot Site 2 (see Figure 6), the scores indicate generally positive task experiences. However, the higher standard deviations suggest greater variability in participant responses. For Pilot site 3, results per user are provided in Figure 7. These scores show an ascending trend in satisfaction across tasks, with Task 3 achieving the highest mean. Task 1 had the lowest score and highest variability, possibly due to interface design challenges mentioned earlier in the manuscript.

It is noted that users were asked to rate each task as a whole rather than each sub-task separately.

**Figure 8:**
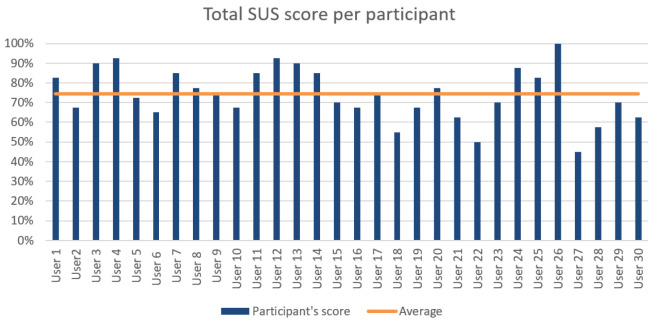
Total SUS score per participant.

System usability. The SUS questionnaire findings were also very encouraging. The average SUS score was 74.43% (Figure 8), exceeding the required acceptability threshold of 70% [90]. Thus, the results indicate that the system usability scored a B grade (i.e., between 74.1% and 77.1%) [89], indicating satisfactory performance. Table 8 summarizes the average score (M) and Standard Deviation (SD) results from the SUS questionnaire for each pilot site and overall.

User impressions. On the final phase of the evaluation, there was a semi-structured interview with each participant. These interviews were very informative concerning their opinions on the system and provided useful information for future improvements. Common findings for all the systems are that:

- Users expressed that they were satisfied with the system.
- Only two felt nausea while wearing the AR glasses.
- The vast majority of users (93%) claimed that the system would be useful in assisting them to become more aware of the situation at hand, given the instant feedback and the clear UI.
- The majority of users (70%) highlighted that they would use it in their daily operations.

However, 10% of participants suggested that they would prefer using the equipment they already have, considering its high precision, whereas 33% of the participants also pointed out that the specific AR glasses were not ergonomic and comfortable to wear throughout their entire work shift; therefore, they would prefer to not use the system in their daily operations. Finally, 6% of the participants do not find the system useful in their daily operations, compared to their existing equipment which they would not like to have substituted. Attributes of the system that were praised by the users were the following:

- The precision of gesture recognition;
- The simplicity of the interaction method;
- The high resolution and clear view of the AR glasses;
- The detailed information provided by the system;
- The real-time information provided all at once;
- The clarity and simplicity of the User Interface;
- The innovative technology;
- The ease of learning concerning the system;
- The prevention of dangers.

Pertinent quotes from what users said are *“Well done! The gestures work flawlessly. This is very important to allow me to focus on my job”; “I like the information that comes all at once right away when something happens with the production line”; “The visuals are clear and the graphics are nice”; “I liked that you can perform the tasks keeping a safe distance from the machine”.*

On the other hand, features that users did not like were the following:

- The flickering of the GUI during the inspection process;
- The AR glasses used which felt uncomfortable for long-term usage;
- The blurry image of the message caused by inappropriate placement of the AR headset;
- The rather small font size of information regarding the detected defects;
- The extra step to perform thumbs-up to start the inspection;
- The defect is obvious so it is pointless to have the system detect it but for more difficult defects it would have potential (from the antenna case study);
- The detections were not visualized instantly on the AR glasses.

Suggestions made by the participants were to:

- ‘Lock’ detections at a certain position and not move along with the user’s field of view, to avoid the flickering effect caused by the continuous change of location of the inspection target;
- Avoid highlighting materials which are good, in the microchip case study, and indicate to the user only materials which are rejectable or borderline acceptable;
- Add Key Performance Indicators (KPIs) that a product should reach;
- Provide statistics pertaining to the production line, such as percentage of defective products identified over the worker’s shift;
- Wireless AR glasses;
- Guidance through animation on how to perform the gesture;
- Voice commands could be better;
- The system should support multiple languages.

Discussion. This user study aimed to assess the usability and the efficiency of the proposed GUI widgets library as well as the intuitiveness of the gestures integrated in the system. The aim of the study was to determine how easy and user-friendly the system is for an operator to use, whether it supports them in their daily job tasks and how satisfied the users are with it. We observed their behavior with the system as well as their answers to the questionnaires and their comments. This way we were able to answer these questions and find out the limitations and problems of the system. Regarding the usability aspect, most users were able to complete the tasks in the scenarios, exhibiting a task performance of 83.7% (70% successful completion on average for Pilot Site 1, 87.1% for Pilot Site 2, and 94% for Pilot Site 3). The users themselves found the tasks rather easy to accomplish, with an average of 5.77 (5.67 for Pilot Site 1, 5.70 for Pilot Site 2, and 5.95 for Pilot site 3) on a scale from 1 (very difficult) to 7 (very easy), thus yielding a rate of 82.42% for ease of use. An analysis of the reasons that led to the partially successful or unsuccessful delivery of some tasks, as well as elaboration on the feedback provided by users through the interview, highlighted five main strands:

1. The learnability of the system without prior training;
2. The clarity and readability of the UI;
3. Barriers introduced by the dynamic nature of the system;
4. Difficulty in recalling and executing multiple gestures;
5. The language of the system.

In more detail, it turned out that even after the short demonstration, participants could always recollect the correct gestures to carry out or the meaning of specific icons. Nevertheless, the system is not intended to be a ‘walk-up-and-use’ system; instead, extensive training is expected to be provided before its actual usage in manufacturing operational environments. Furthermore, some users advocated for incorporating voice commands as an alternative, citing their ease of memorization. However, deploying this feature presents challenges, as certain pilot sites operate in noisy environments, while others require complete silence. In terms of readability, it was concluded that, especially for first-time users, it would be preferable to employ a larger font size, and position UI elements closer to the screen’s center, changes that are going to be introduced to the pertinent widgets and proposed library. In addition, an important finding is that although the system has the potential to provide real-time feedback and visualize defect information on top of a physical object, users suggested to ‘lock’ the visualization in a specific place and avoid a real-time rendering of annotations adapted constantly to the user’s field of view. The option for locking and unlocking the visualization is also an imminent future extension of the widget library. Finally, regarding the ability of the system to support multiple languages, it is a feature that is going to be integrated into the next version of the system.

In terms of suitability for supporting users in their daily job tasks, conclusions can be drawn on the one hand from the task success performance achieved, as discussed above, as well as from participants’ pertinent responses to the corresponding interview question. In this respect, it is notable that 86% of the participants suggested that they find the system useful in becoming more aware of a situation at hand they may be facing, while 70% indicated that they would use it in their daily job operations. Considering that the system is a research prototype, the results are very encouraging and promising for the potential of such context-aware adaptive AR technology for enhancing factory workers’ situational awareness for zero-defect operations. An analysis of users’ responses that were negative indicated that concerns reflect two important aspects that are of general research interest and extend beyond the scope of the current work:

1. The comfortability of the AR device, especially in the context of a full working shift;
2. The trust of operators regarding new technologies and their reluctance to abandon their existing tools and approaches.

With regard to the first, additional studies that range beyond the current work need to be conducted. At the same time, it is hoped, that with advancements in hardware, AR glasses in the future will become even more lightweight and comfortable. Regarding the latter, this is currently an active area of research in the field of explainable AI, but also reflects concerns explored in the literature regarding the adoption of technological innovations in the workplace. Findings from relevant studies [91] indicate that resilience and opportunities for information and training are key ingredients for technology acceptance; considering therefore that the current evaluation was conducted without extensive information and training beforehand, it can be concluded that concerns expressed by workers are natural and expected.

Regarding the third research question, the satisfaction of participants with the system, analysis of the SUS score, and participants’ feedback during the interview yielded positive results. In this regard, the participants expressed positive feedback regarding the system’s user-friendliness, intuitiveness, and ease of learning, particularly regarding the level of granularity and feedback available in a single snapshot of the application. However, there were findings regarding shortcomings of the system based on the users’ opinions, as these have already been analyzed regarding usability improvements. Additional features were also suggested by participants and will be considered in future versions of the system. To summarize the findings from the evaluations based on our observations and the users’ feedback, it is believed that the system exhibits significant potential in assisting users in identifying and rectifying defects within production lines. Despite the majority of users lacking training and exposure to the specific system, they were able to complete the required scenarios successfully, demonstrating the system’s ease of learning and intuitive interface. Both experienced and inexperienced operators were able to utilize the system and carry out the necessary tasks, although it was noted that some users required additional time to become familiar with the gesture-based interface. This study is subject to two main limitations. Firstly, due to the in situ testing of the applications in only three manufacturing pilot sites, the number of users involved in the evaluation was limited. Secondly, the study lacked a cognitive walkthrough for the other two developed applications, which could have provided a more in-depth understanding of the cognitive processes involved in using the interface prior to its development. Nevertheless, this was partially compensated by the co-creation workshops that were conducted with representative end-users, and allowed our understanding of the context of user and end-user requirements.

Guidelines for the design of gesture-based AR systems in industrial settings. This section summarizes the guidelines for the design of gesture-based AR systems, as these were produced based on the systematic review of the literature and complemented with findings from the cognitive walkthrough and user-based evaluation of the applications developed.

(G1) Mid-air gestures are suitable for interactions when the user’s primary focus is not on the system.
(G2) Users exhibit a preference for traditional interactions (e.g., point and click or touch) but the use of hand gestures makes the experience more immersive and enticing for the users, capturing their attention.
(G3) Simple mid-air gestures are easier to remember. When complicated gestures are required, a training phase and a tutorial are beneficial.
(G4) Users have small tolerance for delays and errors in gesture recognition.
(G5) Dynamic gestures have been more widely integrated and tested in the literature; therefore, they constitute an approach that is safe to adopt.
(G6) Consider the gestures that you will employ taking into account the AR technology it is going to use as well as the functionality it needs to provide to the users.
(G7) Follow a Human-Centered Design approach to decide upon the final gesture set, the functionality that the system will offer, as well as the information that it will embed in order to be suitable for the task at hand.
(G8) Avoid overwhelming the user with unnecessary information (e.g., in our case, showing that everything is ok regarding a produced piece of equipment) and extraneous messages (e.g., in our case, different consecutive messages for defect detection and defect predictions).
(G9) When providing information that originates from system reasoning (e.g., information on defect predictions), always provide an account of the underlying rationale.
(G10) When providing information that originates from system reasoning (e.g., information on defect predictions), offer to operators the possibility to optionally verify or contradict the correctness of the system’s decision-making
(G11) Offer the option for ‘locking’ the information visualization to a stable location in the user’s field of view, even if this ‘contradicts’ the dynamic nature and inherent possibilities of the AR system.
(G12) Comfort of the equipment is of paramount importance for its adoption in a working setting.

## 6. Conclusions and Future Work

In this work, we proposed an extended library of GUI components that can be used in context-aware AR applications for decision-making support to operators on the production line. The system provides on-the-fly support and simplifies the process of defect detection and system reconfiguration by taking into consideration various user characteristics, environmental and system factors, and the current task at hand. The AR glasses feature a user-friendly interface and intuitive gestures, which allow operators to easily interact with the system, while maintaining their focus on the task at hand.

For the design and development of the present approach, the Human-Centered Design methodology was followed, which focuses on understanding the needs, wants, and behaviors of people who will use the product or service being designed and actively involving them in the development life-cycle. To gain insight into the contextual factors that require consideration and the users’ requirements, online co-creation workshops were undertaken. The outcomes of these workshops allowed us to design the set of visual components that constitutes the proposed library. The system that we developed notifies the operators about detected or predicted defects and informs them regarding the specific defective product. It also provides guidance in product assembly and step-by-step support in everyday tasks. Finally, the library was integrated into three applications, each of which was designed to address a distinct use case within the manufacturing context.

As per the Human-Centered Design methodology that was employed, the developed applications underwent an evaluation process. To evaluate the proposed solutions, an online cognitive walkthrough was conducted with production line experts, and three in situ end-user evaluations were performed with shop floor operators. Despite minor variations, the primary tasks in all evaluations were consistent: users were required to examine a faulty product and obtain feedback on it and reconfigure the production line. The objective of all evaluations was to assess whether the system is easy to use, if it can effectively and efficiently support operators in their daily job tasks, and whether users are satisfied with the system. The main outcome of all the evaluations is that the system is user-friendly and easy to learn and it can assist users in daily tasks and situations they may be facing. Furthermore, the gestures were found natural, intuitive, and consistent throughout the system. The evaluations revealed the strengths of the proposed approach, but also various issues and limitations associated with the system. Users reported experiencing flickering UI in specific instances and encountering small and blurry text. Furthermore, certain users stated that traditional tools or their special equipment was preferable to the system and that the naked eye could easily detect defects, particularly on antennas, making the inspection process unnecessary for obvious defects. The findings also indicate that certain actions within the system are deemed redundant for experienced users but might be helpful for novice ones. The users also provided valuable feedback regarding features that they considered necessary to be added to the system. Findings from the evaluations have been used to derive design guidelines for gesture-based AR systems in industrial contexts, as well as to identify future improvements of the system.

Regarding future work, we aim at enriching our solution with AI explainability, which is the ability of an AI system to explain its decision-making process and how it arrived at a particular outcome or recommendation [92]. As AI systems become more complex and integrated into industrial settings, it is crucial to understand how they arrive at their decisions, especially when they can have a significant impact on production lines. This is also important for the operators to build trust and confidence in the system, becoming more willing and eager to use it. We are also going to adopt in our approach human-in-the-loop training. Human-in-the-loop training is the iterative process of training a machine learning model using a combination of human guidance and automated algorithms [93]. In the case of Reinforcement Learning, human-in-the-loop training involves a human expert guiding the learning process by providing feedback to the model, which is then used to improve its performance. The operators will be able to provide feedback to each suggestion given by the system with gestures to assist its improvement.

## Figures and Tables

**Table 1:** Ontology Object Properties.

Domains	Object Properties	Ranges
Task	entailsComponent	Component
ProductionLine	consistsOfTask	Task
Component	Employees	ComponentType
Operator	hasComponent	Component
ProductionLine	incorporatesComponent	Component
ProductionLine	initiatesInteraction	Interaction
Operator	performsInteraction	Interaction
Operator	performsTask	Task
ComponentType	providesReaction	Reaction
ProductionLine	requiresReaction	Reaction
Component	supportsInteraction	Interaction
Interaction	triggersReaction	Reaction

**Table 2:** Ontology Data Properties.

Domains	Data Properties	Ranges
Reaction	isActive	Boolean
HUD	hasDisplayHeight	Integer
HUD	hasDisplayWidth	Integer
Component	hasNumberofLoDs	Integer
HUD	hasTileDimention	Integer
Component	hasWidth	Integer
Component	hasHeight	Integer
Component	hasUtility	Decimal

**Table 3:** AR glasses pilot evaluation results for scenario 1.

Screen	Q1	Q2	Q3	Q4	Overall
Defect popup	M: 4.57	M: 5.00	M: 5.00	M: 4.86	M: 4.86
	SD: 0.53	SD: 0.00	SD: 0.00	SD: 0.35	SD: 0.38
Video replay	M: 4.71	M: 5.00	M: 5.00	M: 4.71	M: 4.86
	SD: 0.49	SD: 0.00	SD: 0.00	SD: 0.45	SD: 0.38
Inspection start	M: 4.86	M: 5.00	M: 5.00	M: 4.71	M: 4.89
	SD: 0.38	SD: 0.00	SD: 0.00	SD: 0.45	SD: 0.33
Inspection results	M: 4.57	M: 4.86	M: 4.71	M: 5.00	M: 4.78
	SD: 0.53	SD: 0.35	SD: 0.45	SD: 0.00	SD: 0.44

**Table 4:** AR glasses pilot evaluation results for scenario 2.

Screen	Q1	Q2	Q3	Q4	Overall
Defect popup	M: 4.57	M: 4.86	M: 4.71	M: 5.00	M: 4.78
	SD: 0.53	SD: 0.35	SD: 0.45	SD: 0.00	SD: 0.44
Simulation play	M: 4.71	M: 5.00	M: 5.00	M: 4.57	M: 4.82
	SD: 0.49	SD: 0.00	SD: 0.00	SD: 0.49	SD: 0.41

**Table 5:** Average and standard deviation satisfaction per task for Pilot Site 1 and overall.

	Task 1	Task 2	Task 3	Overall
Average	5.80	5.60	5.60	5.67
SD	0.87	0.92	0.8	0.8

**Table 6:** Average and standard deviation satisfaction per task for Pilot Site 2 and overall.

	Task 1	Task 2	Overall
Average	5.70	5.70	5.70
SD	1.34	1.55	1.40

**Table 7:** Average and standard deviation satisfaction per task for Pilot Site 3 and overall.

	Task 1	Task 2	Task 3	Overall
Average	5.56	5.90	6.40	5.95
SD	1.29	1.19	0.82	1.10

**Table 8:** Average and standard deviation of SUS scores for each pilot site.

	Pilot Site 1	Pilot Site 2	Pilot Site 3	Overall
Average	77.50	77.00	68.8	74.43
SD	0.10	0.11	1.10	0.43

## Data Availability

The data presented in this study are available upon request from the corresponding author due to privacy, legal, and ethical reasons.
